# Absorption kinetics of vitamins and minerals from a novel nutritional product in physically active adults: a randomized, double-blind, placebo-controlled crossover trial

**DOI:** 10.3389/fnut.2026.1793264

**Published:** 2026-07-06

**Authors:** Philip A. Sapp, Jeremy R. Townsend, Trevor O. Kirby, Caitlyn G. Edwards, Michael B. La Monica, Tim N. Ziegenfuss, Matthew J. Vergne, Wendell S. Akers, Ralph Esposito

**Affiliations:** 1 Research, Nutrition, and Innovation, AG1, Carson City, NV, United States; 2 Department of Nutritional Sciences, The Pennsylvania State University, University Park, PA, United States; 3 Health and Human Performance, Concordia University Chicago, River Forest, IL, United States; 4 The Center for Applied Health Sciences, Canfield, OH, United States; 5 Department of Pharmaceutical Sciences, Lipscomb University, Nashville, TN, United States; 6 Department of Chemistry and Biochemistry, Lipscomb University, Nashville, TN, United States; 7 Department of Pharmacology, Vanderbilt University School of Medicine, Nashville, TN, United States; 8 Department of Nutrition, Food Studies, and Public Health, New York University-Steinhardt, New York, NY, United States

**Keywords:** bioavailability, dietary supplements, minerals, pharmacokinetics, vitamins

## Abstract

**Background/objectives:**

Nutrient interactions in multi-ingredient supplements may influence micronutrient absorption and bioavailability, yet pharmacokinetic data exploring these interactions remains generally limited. This clinical trial assessed the acute absorption phase of key micronutrients in AG1, a comprehensive nutritional supplement containing vitamins, minerals, probiotics, and phytochemicals.

**Methods:**

In a randomized, double-blind, placebo-controlled crossover trial 16 healthy adults (8 males and 8 females) consumed a single serving (13 g) of AG1 or a taste- and appearance-matched placebo mixed in water, following a 10-h overnight fast. Each condition was separated by a 1-week washout period. Blood samples were collected 0 (baseline), 30, 60, 90, 120, 180, 240, 360, and 480 min post-ingestion. Plasma concentrations of folate, calcium, zinc, vitamin C, biotin, nicotinamide, pyridoxine, riboflavin, thiamine, and hesperidin were measured. The absorption phase was generally characterized using area under the curve (AUC_0-480 min_), C_max_, and T_max_. Safety and tolerability were assessed throughout the study. Statistical analysis included repeated-measures ANOVA and paired t-tests.

**Results:**

AG1 significantly increased AUC_0-480 min_ values (*p* < 0.05) for all measured nutrients except pyridoxine which revealed a weak trend (*p* = 0.1205). Both AG1 and placebo were well tolerated, with no serious adverse events reported.

**Conclusion:**

Acute consumption of AG1 resulted in measurable increases in circulating levels of most of the tested micronutrients, indicating effective absorption. These findings suggest that AG1 may influence nutrient status in healthy adults.

**Clinical trial registration:**

Clinicaltrials.gov, identifier NCT06316700.

## 1 Introduction

Dietary supplements are widely consumed in the United States, with more than half of adults (61.4%) reporting use (1). The most commonly consumed dietary supplement category is multivitamin–mineral supplements (MVMs), or supplements that contain three or more vitamins and one or more minerals (1). Data from the National Health and Nutrition Examination Survey (NHANES) reveals that dietary supplements are often consumed by US adults with the intent to “improve” (45% of adults) or “maintain” overall health (33%), with MVMs specifically taken by many to “supplement the diet” (2, 3). Evidence consistently supports the ability of MVM supplements to help meet nutrient recommendations and improve indices of nutrient status (4–7). However, recent work by Tinsley et al., identified persistent micronutrient gaps in healthy, physically active adults, with a substantial proportion failing to meet estimated average requirements for several vitamins and minerals, particularly vitamins C, D, E, and folate, despite the perception that this population consumes high-quality diets (8).

Despite widespread MVM use, their demonstrated ability to improve dietary nutrient adequacy, and prevalence of nutrient inadequacies in physically active individuals, there remains a notable lack of data on the absorption kinetics and bioavailability of their vitamins and mineral constituents, both individually and in combination within the MVM matrix. The lack of these data were identified as a key area requiring further research by the 2010 Dietary Guidelines Advisory Committee (9). The committee called for the need to “develop accurate composition and bioavailability data across the multitude of vitamin, mineral, and nutrient supplements, and evaluate outcomes based on nutrient composition and bioavailability within the MVM matrix.” In response, a review by Comerford et al. (10) highlighted the challenges associated with each of these research areas identified in the 2010 DGAC report and the limitations of their referenced studies, suggesting the need for more clinical trials assessing the absorption kinetics of the vitamins and minerals in MVMs and evaluating the bioavailability of MVMs in different forms. However, the review emphasized the complexity and nuances of assessing the bioavailability or pharmacokinetics of MVMs compared to pharmaceuticals given their differences in composition and metabolism (10).

Despite the call for more research from the 2010 DGAC committee, published data on the absorption kinetics of multi-ingredient formulations remain limited. A small number of bioavailability studies assessing MVMs exist (11–13). However, more recent additions to the literature focused on absorption kinetics continue to be scarce (14–17). The complexity of the MVM matrix and inclusion of other bioactives in multi-ingredient products introduce multiple factors that may influence nutrient absorption. For example, nutrient-nutrient (18) and nutrient-matrix (19) interactions can modulate nutrient absorption and bioavailability, underscoring the need to examine how ingredient combinations and matrix composition influences *in vivo* absorption.

The multi-ingredient dietary supplement, AG1, is a nutritional supplement that combines vitamins, minerals, phytonutrients, prebiotics, and probiotics, with whole food powders and other similar ingredients. Within complex matrices like these, it is unknown if certain vitamins and minerals exhibit delayed, impaired, or improved absorption due to physical and chemical interactions, prompting the need to characterize the absorption kinetics of key nutrients in full formulations. A prior formulation of AG1 was assessed using the Simulator of the Human Intestinal Microbial Ecosystem® (SHIME), which demonstrated that its minerals were bioaccessible and likely to enter systemic circulation *in vitro*. However, the model’s constraints limited accurate assessment of a wider array of nutrients which are more appropriately measured *in vivo* (20). Thus, validating the plasma appearance and kinetic parameters of AG1’s nutrients is essential to substantiate its supplemental efficacy.

Therefore, the present study was designed to quantitatively assess the plasma appearance and absorption kinetics of key nutrients in AG1. We conducted a double-blind, randomized, placebo-controlled crossover trial in healthy men and women to measure the plasma appearance of several vitamins and minerals from AG1. Primary outcomes included measuring plasma appearance for folate, calcium, zinc, and vitamin C. Secondary outcomes consisted of additional pharmacokinetic outcome variables for these nutrients. Exploratory outcomes consisted of B-vitamins, tolerability, and safety. By characterizing the plasma appearance of these nutrients over an 8-h period post-consumption, we aimed to evaluate some of the absorption kinetics of AG1 and contribute to the limited body of literature on multi-ingredient supplement pharmacokinetics. This study addresses a gap in pharmacokinetic data of complex formulations and evaluates whether AG1 can effectively deliver bioavailable nutrients via its MVM matrix.

## 2 Materials and methods

### 2.1 Participants

Sixteen healthy men (n = 8) and women (n = 8) completed this trial. Eligible participants were enrolled if they were recreationally active (exercised at least 3 times per week) and in good health based on their health history, physical examination, and blood chemistries. Participants were ages 18–45 years, had a body mass index of 18.5–29.9 kg/m2, had normotensive blood pressure (systolic ≤140 mm Hg and diastolic ≤ 90 mm Hg), had a seated resting heart rate ≤ 90 beats per minute, and were willing to duplicate their baseline dietary intake, refrain from caffeine and exercise for 24 h prior, and fast for 10 h prior to each testing visit. Before enrollment, all participants indicated their willingness to comply with all aspects of the experimental protocol. Exclusion criteria included: (a) MVM supplementation within the past 3 months or any other dietary supplement which would interfere with study outcomes (e.g., vitamins, minerals, probiotics, prebiotics, etc.) (b) subjects who have received an antibiotic within 3 months prior to study entry or current use of prescription or OTC medications that could influence study outcomes; (c) presence of chronic disease or medical condition such as cancer, gastrointestinal, cardiovascular, respiratory, liver, renal, thyroid, autoimmune, or metabolic conditions; (d) alcohol consumption (more than 2 standard alcoholic drinks per day or more than 10 drinks per week), drug abuse or dependence within the past 6 months; (e) current smokers, vapers, or tobacco users or use of tobacco within the past month (f) pregnant women, women trying to become pregnant, women less than 120 days postpartum or nursing women. (g) a change in hormone therapy, including oral contraceptives, within 4 weeks prior to screening, or unwilling to maintain current hormone therapy/oral contraceptive use throughout the course of the study; (h) known sensitivity to any ingredient in the test formulations as listed in the product label. Participants were also excluded if they were currently participating in another research study with an investigational product or have been in another research study in the past 30 days, subject adhered to a specialty diet including but not limited to, Vegan, Vegetarian, Ketogenic, Paleo, Atkins, South Beach, Carnivore, etc.; or had donated blood or plasma within the previous week.

This study was conducted according to the guidelines in the Declaration of Helsinki of 1975, and all study procedures were approved by the WCG Institutional Review Board (#AG-02-0124). Prior to enrollment, written informed consent was obtained from all study participants.

### 2.2 Experimental design

**Figure 1:**
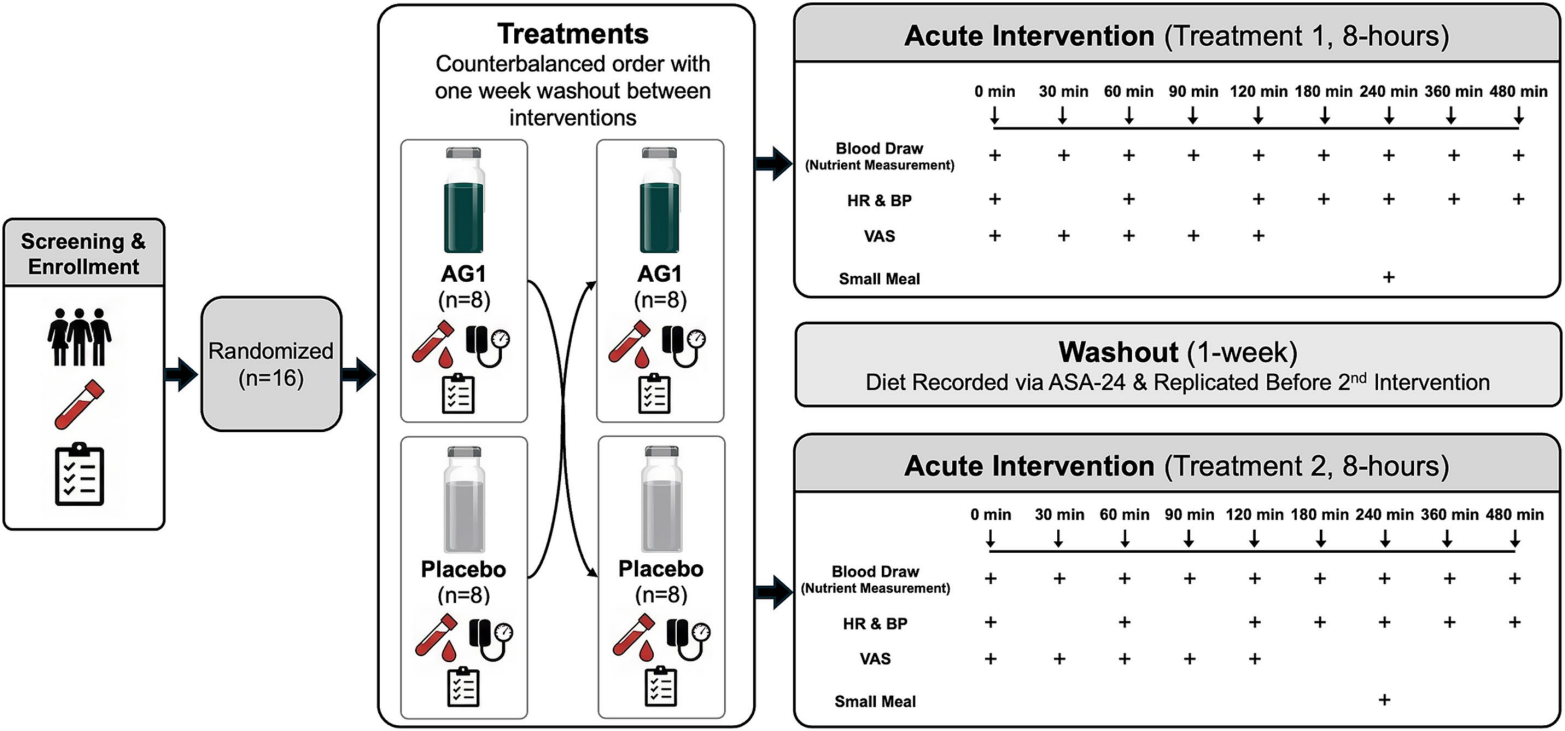
Study diagram detailing the study flow for subjects participating in this study. BP, blood pressure; HR, heart rate; min, minutes; and VAS, visual analog scale.

This was a double-blind, randomized, placebo-controlled crossover clinical trial. Participants attended three laboratory visits throughout the study: a screening visit and two testing visits to complete the pharmacokinetic assessments on AG1 and placebo (PL) (Figure 1). AG1 and PL conditions were separated by a washout period of at least 1 week. Participants were randomized to their respective treatment sequence. Both participants and investigators were blinded to the study products. This study was registered on clinicaltrials.gov (NCT06316700) and first posted on December 26th, 2023.

### 2.3 Interventional products

This study assessed the acute absorption kinetic parameters of one dose of AG1 compared to a taste and color matched placebo. Participants orally ingested AG1® (13 g; AG1, Carson City, NV, USA) or PL (13 g maltodextrin + flavoring). The same flavoring was used in both interventional products. The nutritional information and ingredients for AG1® are presented in Supplementary Table S1 and the full product has undergone evaluation and verification via NSF testing (Ann Arbor, MI, USA) to ensure the product meets strict quality, purity, safety, and label accuracy standards. Both interventional products were prepared (mixed in 10 ounces of water) by the study staff and presented to the study participants during their testing visits. As previously stated, study products were blinded to participants and study personnel.

### 2.4 Study procedures

Participants were asked to refrain from exercise, alcohol, and caffeine intake for 24 h and complete a 10-h overnight fast before each study visit. At the screening visit, participant’s height (wall mounted stadiometer), weight (Seca™ Medical Scale), vitals (heart rate and blood pressure; Omron HEM907XL), medical history, baseline dietary intake (Automated Self-Administered 24-Hour Dietary Assessment Tool, National Cancer Institute, Rockville, MD), and blood work (lipid panel, CBC with differential, and CMP) was assessed. To replicate baseline-testing conditions as closely as possible, subjects were provided with a copy of their initial dietary record. Prior to each subsequent visit to the laboratory, subjects followed their diet in accordance with the provided copy of their initial dietary record as confirmed by review of dietary records. During visits 2 and 3, participants’ weight and dietary intake was assessed prior to beginning the pharmacokinetic protocol. During visits 2 and 3, blood nutrient concentrations were measured at baseline, 30, 60, 90, 120, 180, 240, 360, 480 min post ingestion. Vitals (heart rate and blood pressure) were assessed at baseline, 60, 120, 180, 240, 360, 480 min post-ingestion during visits 2 and 3. Visual analog scales were completed by participants during visits 2 and 3 at baseline, 30, 60, 90, 120 min post-ingestion. After the four-hour time point measurements, subjects consumed a small, standardized meal. This meal was prepared by study personnel and consisted of 1 cup of Minute white rice (76 g total carbohydrates and 8 g protein) and 3 ounces of grilled chicken (2 g total fat, 60 mg cholesterol, 420 mg sodium, 3 g total carbohydrates, 29 g protein, and 288 mg potassium).

### 2.5 Blood sample collection and handling

Blood draws took place during each study visit. At screening a single blood draw was collected with a total blood volume of approximately 12 mL. During visits 2 and 3 serial blood draws were completed using an intra-venous catheter that was inserted into an upper extremity vein before and 30-, 60-, 90-, 120-, 180-, 240-, 360-, and 480 min post ingestion of the investigational product. The blood handling procedures were as follows at each timepoint: 1 serum separator tube (allowed to clot for 30 min and spun in LabCorp centrifuge then sent to LabCorp for calcium and folate analysis), 1 large heparin coated tube (immediately spun twice in LabCorp centrifuge, split into 4 separate 0.5 mL aliquots, stored at −80 degrees Celsius), 1 small heparin coated tube (immediately spun in LabCorp centrifuge, aliquoted 2 mL into dark cryovial, stored for >24 h at −80 degrees Celsius, and sent to LabCorp for vitamin C analysis), and 1 trace element K2 EDTA tube (immediately spun in Eppendorf for 15 min at 3,000 RPM and 20 degrees Celsius, aliquoted 2 mL to metal free transfer tube, and sent to LabCorp for zinc analysis). Additional aliquots for analysis of the remaining blood markers were stored at −80 degrees Celsius and were analyzed after the completion of the study.

### 2.6 Laboratory analysis of analytes

A subset of the prepared blood specimens were submitted to LabCorp (Dublin, OH, USA) for the lipid panel (Test Number 303756), CBC with differential (Test Number 005009), comprehensive metabolic panel (Test Number 322000), total serum folate (Test Number 002014), total serum zinc (Test Number 001800), total plasma vitamin C (Test Number 001805), and total serum calcium (Test Number 001016) analysis (21–27). The lipid panel included total cholesterol, HDL cholesterol, LDL cholesterol, triglycerides, and VLDL cholesterol. The CBC with differential panel included hematocrit, hemoglobin, mean corpuscular volume (MCV), mean corpuscular hemoglobin (MCH), mean corpuscular hemoglobin concentration (MCHC), red cell distribution width (RDW), percentage and absolute differential counts, platelet count (PLT), red cell count (RBC), and white blood cell count (WBC). The comprehensive metabolic panel included alanine aminotransferase (ALT/SGPT), albumin:globulin (A: G) ratio, serum albumin, serum alkaline phosphatase, aspartate aminotransferase (AST/SGOT), total bilirubin, BUN, BUN:creatinine ratio, serum calcium, total carbon dioxide, serum chloride, serum creatinine, eGFR calculation, total globulin, serum glucose, serum potassium, total serum protein, and serum sodium. All lab analyses were conducted using LabCorp’s standard operating procedures, with more information available through their website.

The remaining blood specimens were submitted to Lipscomb University (Nashville, TN, USA) to measure serum levels of a vitamin B panel, serum phytochemicals, and total antioxidant capacity. The serum vitamin B panel consisted of thiamine, biotin, riboflavin, nicotinamide, and pyridoxine. The phytochemical panel consisted of hesperidin, a citrus bioflavonoid. Calibration and QC standards were prepared in concentrations of 0.1 ng/mL to 1,000 ng/mL for the mixture of each vitamin and citrus bioflavonoid (Supplementary Table S2) (28). Internal standard calibration curves were prepared for each analyte.

A simple protein precipitation procedure was employed for sample preparation. To 100 μL of plasma sample (calibration standards, QC samples, or study samples), 300 μL of internal standard solution (100 ng/mL internal standard mix in acetonitrile) was added in a well on a 96-well protein precipitation plate (Restek Resprep PPT3, Bellefonte, PA). The mixture was vortexed for 30 s, and positive pressure was used to collect filtrate into a 96-well deep well sample plate. The filtrate was dried with a stream of nitrogen, reconstituted with 100 μL of water, and vortexed briefly before being transferred to the LC–MS/MS for analysis.

Chromatographic separation was performed on a Shimadzu Prominence HPLC system (Shimadzu, Columbia, MD) equipped with a binary pump, autosampler, and column oven. Mass spectrometric detection was carried out using a Shimadzu LCMS 8040 triple quadrupole mass spectrometer equipped with a Dual Electrospray/Atmospheric Pressure Chemical Ionization (DuIS). Chromatographic separation was achieved on a Restek Aqua Ultra Aqueous C18 column (50 × 2.1 mm, 3 μm) maintained at 40 °C. The mobile phase consisted of (A) 0.1% formic acid and 5 mM ammonium formate in water and (B) 0.1% formic acid in 5 mM ammonium formate in acetonitrile. The flow rate was 0.6 mL/min with a total run time of 6 min. The gradient program was as follows: 0.0–2.0 min, 0–65% B; 2.01 min, 90% B; 2.01–4.0 min, 90–95% B; 4.0–4.5 min, 95% B; 4.51 min, 0% B (re-equilibration). The injection volume was 20 μL.

The mass spectrometer was operated in positive ionization (+) mode for all analytes. The optimized source parameters were: Desolvation Line 250 °C, Heat Block 400 °C, and Drying Gas Flow 20 L/min, and Nebulizing gas flow 3 L/min. Compound-specific parameters, including precursor ion, product ion, Q1 pre-rod bias, collision energy (CE), and Q3 pre-rod bias, were optimized for each analyte and internal standard (Supplementary Table S3). The concentrations were determined by comparing the sample peak area ratio to the corresponding calibration curve for each analyte.

### 2.7 Subjective outcome assessments

Participants were asked to complete Visual Analog Scales (VAS) during visit two and three at baseline, 30-, 60-, 90-, and 120-min post-ingestion of the investigational product. These were used to assess levels of bloating, hunger, flatulence (gas), fullness (satiety), energy, sleepiness, desire to eat (appetite), prospective food consumption, and overall mood. A standardized 10 cm line was used with the left end of each line anchored with descriptors depicting the lowest possible level/score and the highest possible level/score descriptors were anchored to the right of each line. Following data collection, the VAS responses were quantified by measuring the distance from the zero anchor to each participant’s mark in millimeters (range: 0–10 cm for each metric).

### 2.8 Pharmacokinetic calculations

The key pharmacokinetic parameters measured included maximum plasma concentration observed (C_max_), time to maximum plasma concentration was observed (T_max_), and plasma exposure over 8 h. Maximal concentration was determined by using the highest observed concentration, with the time to maximum concentration simply being the time point in which C_max_ was observed. Plasma exposure was quantified as the total area under the concentration-time curve from 0 to 480 min (AUC_0-480 min_), calculated utilizing the linear trapezoidal rule (GraphPad Software version 10.3.1 for Windows, Boston, Massachusetts USA). AUC_0-480 min_ was used as the primary measure of systemic nutrient exposure. Because several nutrients have endogenous baseline concentrations, incremental AUC (iAUC_0-480 min_) was additionally calculated as a sensitivity analysis. For these analyses, the baseline concentration of each respective nutrient was subtracted from all post-dose concentrations prior to AUC calculation.

### 2.9 Safety monitoring and adverse events

All local and systemic non-serious and serious adverse events (AEs) were reported by the investigators, assessed, and coded using the Medical Dictionary for Regulatory Activities (MedDRA). The intensity of an AE was subsequently graded according to the protocol-defined criteria based on the Common Terminology Criteria for Adverse Events (CTCAE) Version 5.0, 2017.

### 2.10 Statistical analyses

Based on previous pharmacokinetic studies examining these nutrients of interest, a minimum sample size of *N* = 10 was chosen to detect statistically significant differences between treatments in total area under the concentration-time curve AUC_0-t_, which was prespecified as the primary outcome measure for folate, calcium, zinc, and vitamin C. Sample size estimation was conducted using G*Power (version 3.1) assuming a two-tailed paired t-test, alpha of 0.05 and power of 0.80. Because directly comparable crossover studies evaluating total plasma vitamin/mineral AUC following multivitamin versus placebo administration are limited, the effect size assumption was informed by the closest available folate pharmacokinetic crossover data (29). In that study, plasma folate differed by a mean of 10.6 units between the experimental condition in 16 subjects (paired t-test *p* < 0.0001), corresponding to a conservative standardized within-subject effect size (Cohen’s dz. ~ 1.3). Because the present study uses a randomized balanced two-period crossover design in which each participant serves as their own control, power calculations were based on the within-subject paired comparison of AUC values. This analysis indicated a sample size estimate of 5 participants per sex. Therefore, we aim to enroll 8 men and 8 women for dropouts and participant variability.

Folate, calcium, zinc, and vitamin C AUC_0-480 min_ were prespecified as the primary endpoints. C_max_ was prespecified as a secondary outcome variable for the primary nutrients. T_max_ was reported as a descriptive characterization of absorption kinetics. Additional analytes included other B-vitamins, hesperidin, and nutrient concentrations at individual sampling timepoints, were evaluated as exploratory endpoints to further characterize absorption kinetics. Tolerability and safety measures were also assessed for both treatment conditions. Baseline-adjusted AUC (iAUC_0-480 min_) was evaluated as a sensitivity analysis.

Descriptive statistics (mean and SD) were used to quantify subjects’ baseline physical characteristics as well as their clinical chemistry and hematological biomarkers. Urine specific gravity, body weight, and dietary intake at baseline for both conditions were assessed with paired t-tests.

For summary pharmacokinetic parameters, treatment effects comparing AG1 and placebo were assessed using within subject paired comparisons (paired t-tests) consistent with the crossover design for AUC_0-480 min_ and C_max_. T_max_ was reported descriptively for the AG1 group arm only, and only for analytes demonstrating a statistically significant C_max_ difference relative to placebo, as T_max_ meaningfully characterizes an absorption-drive peak only when such a peak has been established; T_max_ was not inferentially compared between conditions. Two-way repeated-measures ANOVA with treatment, time, and time x treatment interaction effects was utilized to compare the concentrations of each nutrient at each time point between AG1 and the placebo group. When significant interaction effects were observed, Sidak *post hoc* comparisons were performed. Potential period and sequence effects were evaluated to assess possible carryover.

To account for multiplicity across the four primary endpoints, *p*-values were adjusted using the Benjamini-Hochberg false discovery rate (FDR) procedure. All other analytes and timepoint-specific comparisons were considered exploratory and presented descriptively without adjustment for multiple testing. For p-values reported as thresholds (e.g., “*P* < 0.001”), a conservative value equal to the threshold was used for FDR adjustment.

Visual analog scale response, blood pressure, and heart rate are presented as mean ± SD and were analyzed using two-way repeated-measures ANOVA to compare measurements across time between the AG1 and placebo conditions. All variables were tested for normality using results from a Shapiro–Wilk test. In instances where normality assumptions were not met, nonparametric tests (e.g., Wilcoxon signed-rank tests) were used, and these analyses are indicated where applicable. For all statistical tests, significance was set at *p* < 0.05. All statistical analyses were conducted using GraphPad Prism version 10.3.1.

## 3 Results

### 3.1 Participants

**Figure 2:**
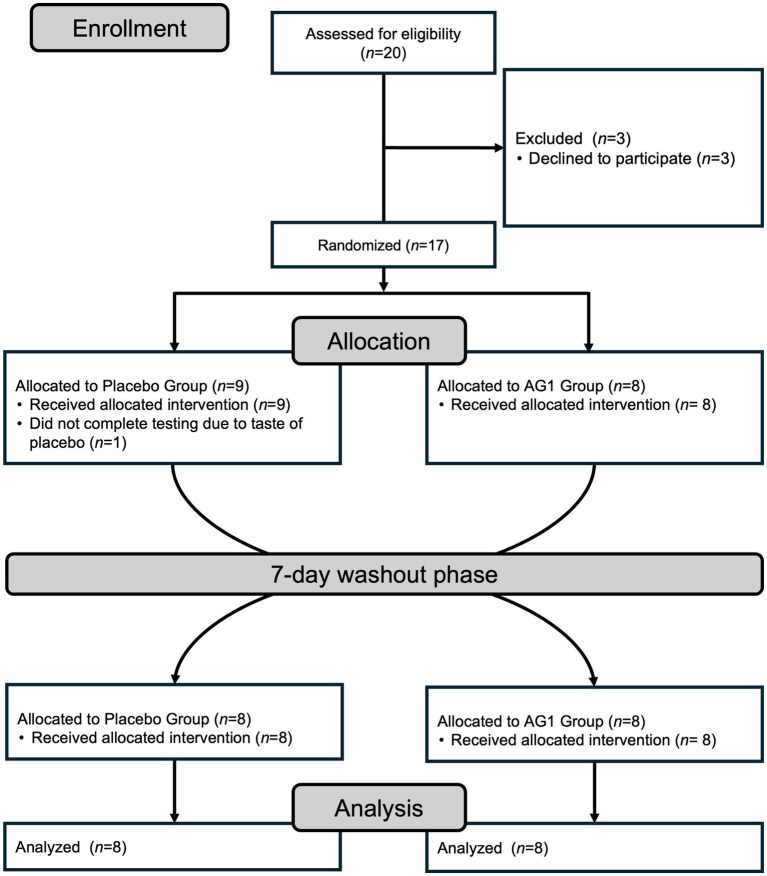
CONSORT flow diagram.

Of the 17 subjects enrolled, 16 enrolled participants completed the study (Figure 2). Participants consisted of 8 healthy males (27.4 ± 8.9 yrs.; 81.5 ± 11.8 kg) and 8 healthy females (33.1 ± 9.7 yrs.; 68.9 ± 6.0 kg). Baseline characteristics, anthropometrics and blood chemistry values are presented in Table 1 and Supplementary Table S4.

**Table 1:** Baseline demographics and clinical characteristics of the study participants.

Variable	Total (*n* = 16)	Males (*n* = 8)	Females (*n* = 8)
Age (years)	30.3 ± 9.5	27.4 ± 8.9	33.1 ± 9.7
Height (cm)	171.4 ± 10.0	178.8 ± 7.8	164.0 ± 5.5
Weight (kg)	75.2 ± 11.1	81.5 ± 11.8	68.9 ± 6.0
Body mass index (kg/m^2^)	25.2 ± 2.0	24.7 ± 1.9	25.8 ± 2.1
Systolic blood pressure (mm Hg)	116.4 ± 13.7	121.1 ± 14.0	111.6 ± 12.3
Diastolic blood pressure (mm Hg)	71.3 ± 6.9	68.4 ± 5.5	74.1 ± 7.3
Resting heart rate (bpm)	67.6 ± 14.2	62.3 ± 13.0	72.9 ± 14.0

Values are presented as mean ± SD for the total sample (*n* = 16). Bpm, beats per minute; Cm, centimeters; kg, kilograms; kg/m^2^, kilograms per meter^2^; and mm Hg, millimeters of mercury.

### 3.2 Vitamin and mineral results

#### 3.2.1 Folate

**Figure 3:**
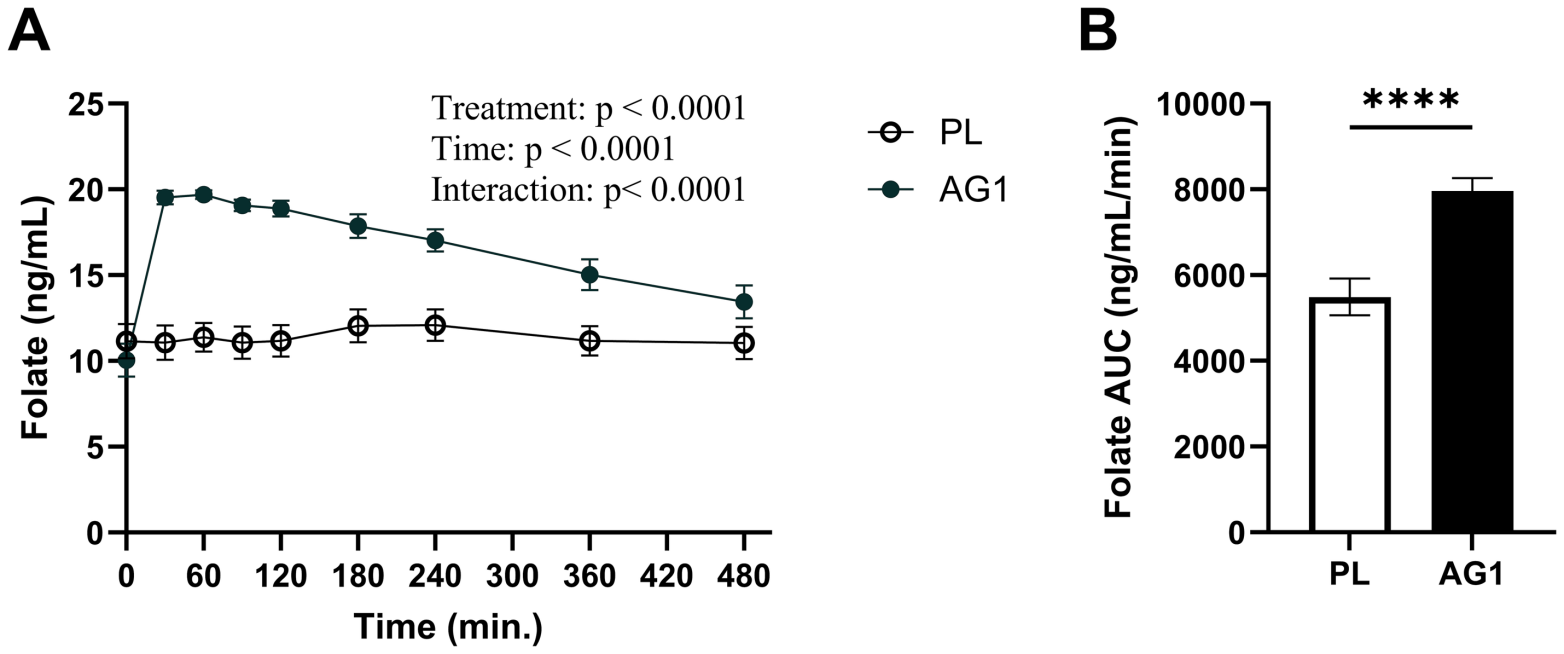
Folate concentrations and total AUC. Visualization of changes in the folate curves over the duration of 480 min **(A)** and total AUC **(B)**. ng/mL, nanogram/milliliter; ng/mL/min, nanogram/milliliter/min; and PL, placebo. Data presented as mean ± SEM. ****p < 0.0001; *n* = 16.

Folate (Figures 3A,B and Supplementary Table S5) concentrations were significantly elevated at 30, 60, 90, 120, 180, 240, 360, and 480 min post ingestion for AG1 relative to placebo. When AG1 was ingested AUC_0-480 min_ and C_max_ were 8,124 ± 1,297 ng/mL/min and 20.8 ± 0.7 ng/mL, respectively, compared with 5,489 ± 1,723 ng/mL/min and 12.9 ± 3.9 ng/mL following placebo ingestion. The within-subject mean difference in AUC_0-480 min_ between AG1 and placebo was 2,635 ng/mL/min (95% CI: 2,106, 3,162; *p* < 0.001) and C_max_ was significantly higher following AG1 ingestion (p < 0.001). T_max_ following AG1 ingestion was 37.5 ± 17.3 min. AUC_0-480 min_ remained significant after Benjamini-Hochberg FDR adjustment (*p* < 0.00013) and sensitivity analysis using iAUC_0-480 min_ yielded similar results (Supplementary Table S5).

#### 3.2.2 Calcium

**Figure 4:**
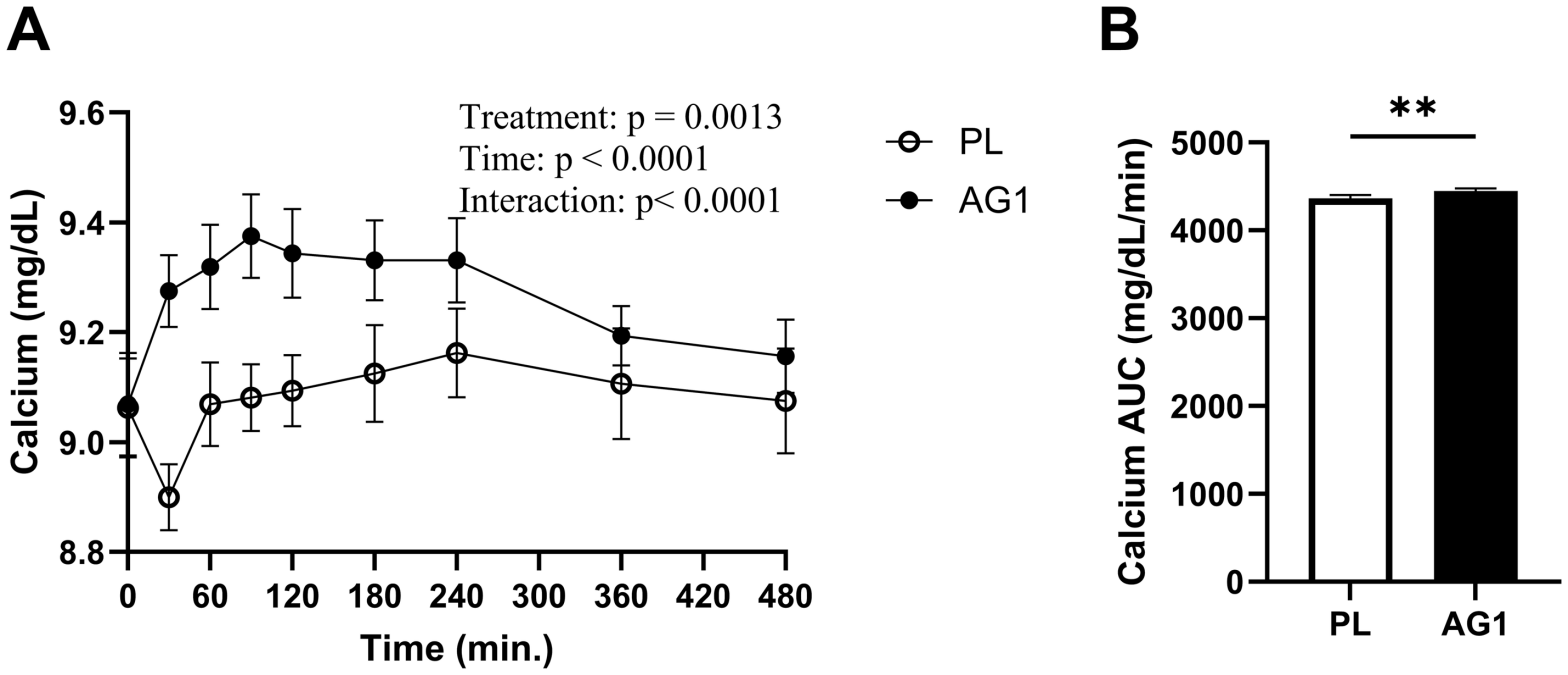
Calcium concentrations and total AUC. Visualization of changes in the calcium curves over the duration of 480 min **(A)** and total AUC **(B)**. mg/dL, milligram/deciliter; mg/dL/min, milligram/deciliter/minute; and PL, placebo. Data presented as mean ± SEM. Total AUC comparisons were performed using Wilcoxon signed-rank test. **p < 0.01; *n* = 16.

Calcium (Figures 4A,B and Supplementary Table S5)concentrations were significantly elevated at 30, 60, 90, 120, 180, and 240 min post ingestion for AG1 relative to placebo. When AG1 was ingested AUC_0-480 min_ and C_max_ were 4,448 ± 116.3 mg/dL/min and 9.5 ± 0.3 mg/dL, respectively, compared with 4,366 ± 145.6 mg/dL/min and 9.4 ± 0.3 mg/dL following placebo ingestion. The within-subject median difference in AUC_0-480 min_ between AG1 and placebo was 86.3 mg/dL/min (Wilcoxon signed-rank test, *p* = 0.0049) and C_max_ was significantly higher following AG1 ingestion (*p* = 0.0468). T_max_ following AG1 ingestion was 129.4 ± 88.8 min. AUC_0-480 min_ remained significant after Benjamini-Hochberg FDR adjustment (*p* = 0.0049) and sensitivity analysis using iAUC_0-480 min_ yielded similar results (Supplementary Table S5).

#### 3.2.3 Zinc

**Figure 5:**
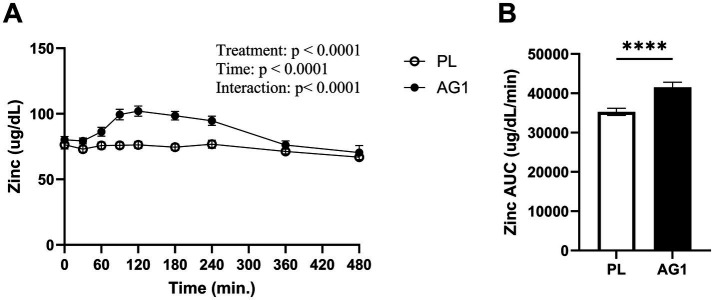
Zinc concentrations and total AUC. Visualization of changes in the zinc curves over the duration of 480 min **(A)** and total AUC **(B)**. ug/dL, microgram/deciliter; ug/dL/min, microgram/deciliter/minute; and PL, placebo. Data presented as mean ± SEM. ****p < 0.0001; *n* = 16.

Zinc (Figures 5A,B and Supplementary Table S5) concentrations were significantly elevated at 60, 90, 120, 180, and 240 min post ingestion for AG1 relative to placebo. When AG1 was ingested AUC_0-480 min_ and C_max_ were 41,140 ± 2,938 μg/dL/min and 111.6 ± 14.0 μg/dL, respectively, compared with 35,688 ± 3,353 μg/dL/min and 87.1 ± 8.8 μg/dL following placebo ingestion. The within-subject mean difference in AUC_0-480 min_ between AG1 and placebo was 5,452 μg/dL/min (95% CI: 4,193, 6,711; *p* < 0.0001) and C_max_ was significantly higher following AG1 ingestion (*p* < 0.0001). T_max_ following AG1 ingestion was 150.0 ± 68.0 min. AUC_0-480 min_ remained significant after Benjamini-Hochberg FDR adjustment (*p* < 0.00013) and sensitivity analysis using iAUC_0-480 min_ yielded similar results (Supplementary Table S5).

#### 3.2.4 Vitamin C

**Figure 6:**
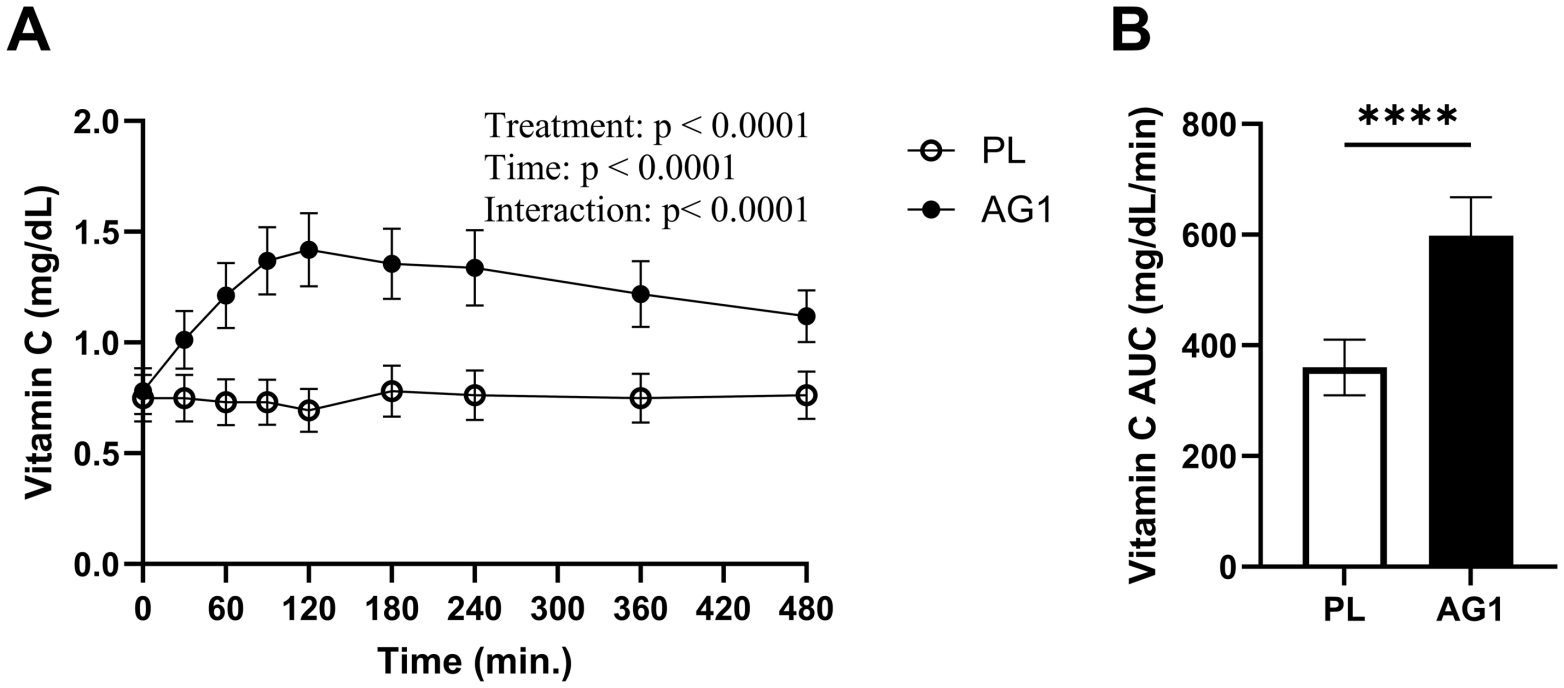
Vitamin C concentrations and total AUC. Visualization of changes in the vitamin C curves over the duration of 480 min **(A)** and total AUC **(B)**. mg/dL, milligram/deciliter; mg/dL/min, milligram/deciliter/minute; and PL, placebo. Data presented as mean ± SEM. ****p < 0.0001; *n* = 16.

Vitamin C (Figures 6A,B and Supplementary Table S5) concentrations were significantly elevated at 30, 60, 90, 120, 180, 240, 360, and 480 min post ingestion for AG1 relative to placebo. When AG1 was ingested AUC_0-480 min_ and C_max_ were 598.4 ± 276.5 mg/dL/min and 1.6 ± 0.7 mg/dL, respectively, compared with 357.7 ± 205.5 mg/dL/min and 0.9 ± 0.5 mg/dL following placebo ingestion. The within-subject mean difference in AUC_0-480 min_ between AG1 and placebo was 240.8 mg/dL/min (95% CI: 172.6, 308.9; p < 0.0001) and C_max_ was significantly higher following AG1 ingestion (p < 0.0001). T_max_ following AG1 ingestion was 146.3 ± 96.0 min. Exploratory sequence analyses indicated that the AG1—Placebo difference for vitamin C was larger in the participants assigned to the AG1 then Placebo sequence compared to those assigned Placebo then AG1 sequence. However, this pattern was not consistent with a standard carryover effect, which typically would attenuate treatment differences in the AG1 then Placebo sequence. AUC_0-480 min_ remained significant after Benjamini-Hochberg FDR adjustment (*p* < 0.00013) and sensitivity analysis using iAUC_0-480 min_ yielded similar results (Supplementary Table S5).

#### 3.2.5 Biotin

**Figure 7:**
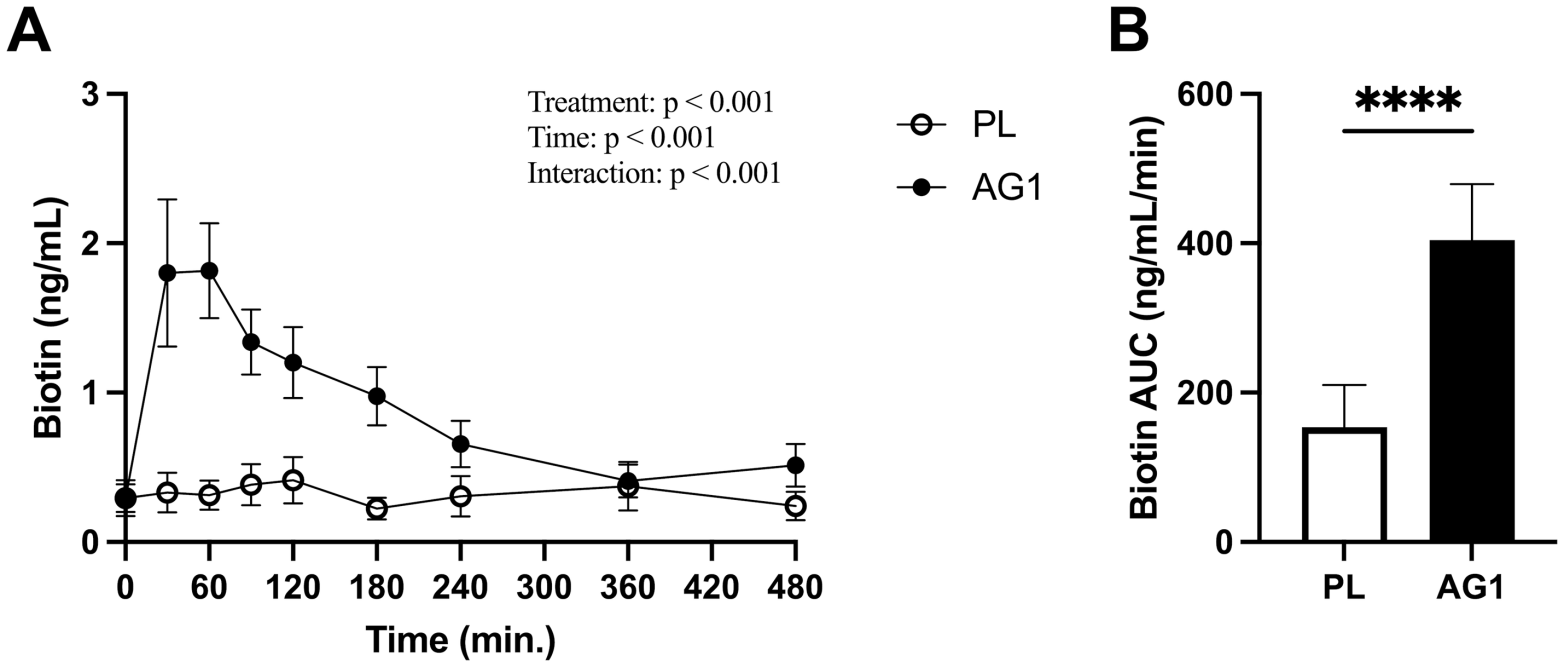
Biotin concentrations and total AUC. Visualization of changes in the biotin curves over the duration of 480 min **(A)** and total AUC **(B)**. ng/mL, nanogram/milliliter; ng/mL/min, nanogram/milliliter/min; and PL, placebo. Data presented as mean ± SEM. ****p < 0.0001; *n* = 16.

Biotin (Figures 7A,B and Supplementary Table S5) concentrations were significantly elevated at 30, 60, 90, 120, and 180 min post ingestion for AG1 relative to placebo. When AG1 was ingested AUC_0-480 min_ and C_max_ were 404.4 ± 298.3 ng/mL/min and 2.3 ± 1.9 ng/mL, respectively, compared with 154.0 ± 225.1 ng/mL/min and 0.6 ± 0.7 ng/mL following placebo ingestion. The within-subject mean difference in AUC_0-480 min_ between AG1 and placebo was 250.5 ng/mL/min (95% CI: 151.8, 349.2; p < 0.0001) and C_max_ was significantly higher following AG1 ingestion (*p* = 0.0004). T_max_ following AG1 ingestion was 58.1 ± 31.9 min. Sensitivity analyses using iAUC_0-480 min_ yielded similar results (Supplementary Table S5).

#### 3.2.6 Riboflavin

**Figure 8:**
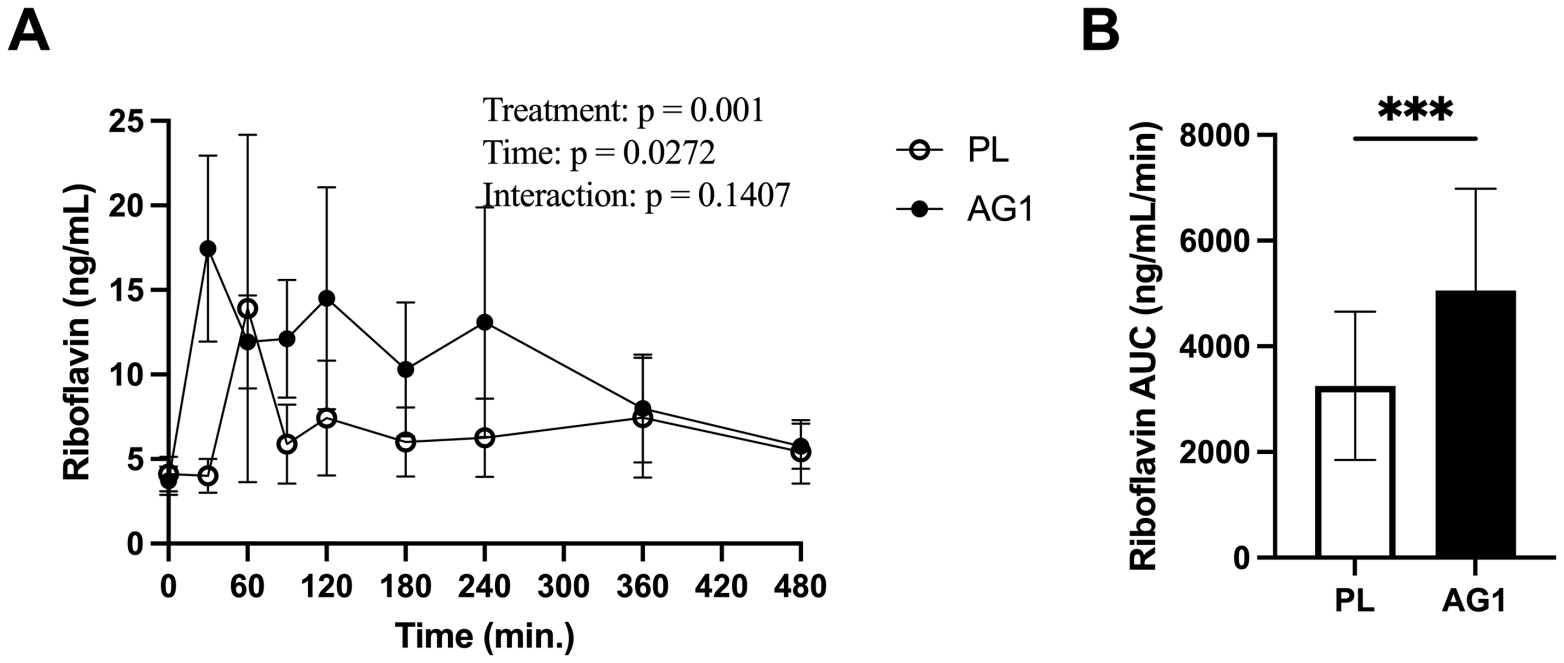
Riboflavin concentrations and total AUC. Visualization of changes in the riboflavin curves over the duration of 480 min **(A)** and total AUC **(B)**. ng/mL, nanogram/milliliter; ng/mL/min, nanogram/milliliter/min; and PL, placebo. Data presented as mean ± SEM. Total AUC comparisons were performed using Wilcoxon signed-rank test. ***p < 0.001; *n* = 16.

Riboflavin (Figures 8A,B and Supplementary Table S5) concentrations were significantly elevated at 30 min post ingestion for AG1 relative to placebo. When AG1 was ingested AUC_0-480 min_ and C_max_ were 5,054 ± 7,711 ng/mL/min and 19.6 ± 25.7 ng/mL, respectively, compared with 3,253 ± 5,626 ng/mL/min and 15.3 ± 40.9 ng/mL following placebo ingestion. The within-subject median difference in AUC_0-480 min_ between AG1 and placebo was 1,260 ng/mL/min (Wilcoxon signed-rank test, *p* = 0.0003) and C_max_ was significantly higher following AG1 ingestion (*p* = 0.0076). T_max_ following AG1 ingestion was 78.8 ± 82.6 min. Sensitivity analyses using iAUC_0-480 min_ yielded similar results (Supplementary Table S5).

#### 3.2.7 Thiamine

**Figure 9:**
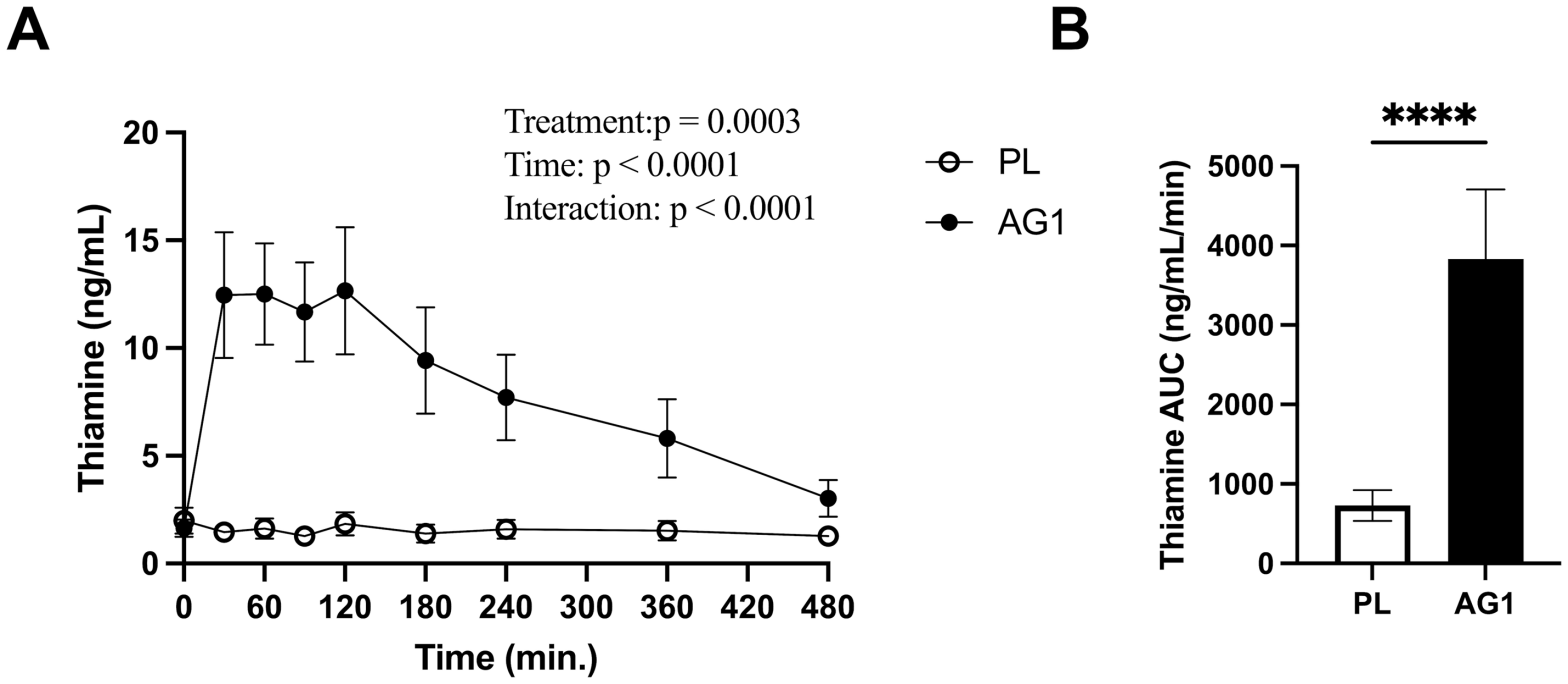
Thiamine concentrations and total AUC. Visualization of changes in the thiamine curves over the duration of 480 min **(A)** and total AUC **(B)**. ng/mL, nanogram/milliliter; ng/mL/min, nanogram/milliliter/min; and PL, placebo. Data presented as mean ± SEM. Total AUC comparisons were performed using Wilcoxon signed-rank test. ****p < 0.0001; *n* = 16.

Thiamine (Figures 9A,B and Supplementary Table S5) concentrations were significantly elevated at 30, 60, 90, 120, 180, 240, and 360 min post ingestion for AG1 relative to placebo. When AG1 was ingested AUC_0-480 min_ and C_max_ were 3,832 ± 3,503 ng/mL/min and 17.4 ± 13.0 ng/mL, respectively, compared with 730.1 ± 771.9 ng/mL/min and 2.3 ± 2.5 ng/mL following placebo ingestion. The within-subject median difference in AUC_0-480 min_ between AG1 and placebo was 1,957 ng/mL/min (Wilcoxon signed-rank test, *p* < 0.0001) and C_max_ was significantly higher following AG1 ingestion (*p* < 0.0001). T_max_ following AG1 ingestion was 84.4 ± 49.3 min. Sensitivity analyses using iAUC_0-480 min_ yielded similar results (Supplementary Table S5).

#### 3.2.8 Pyridoxine

**Figure 10:**
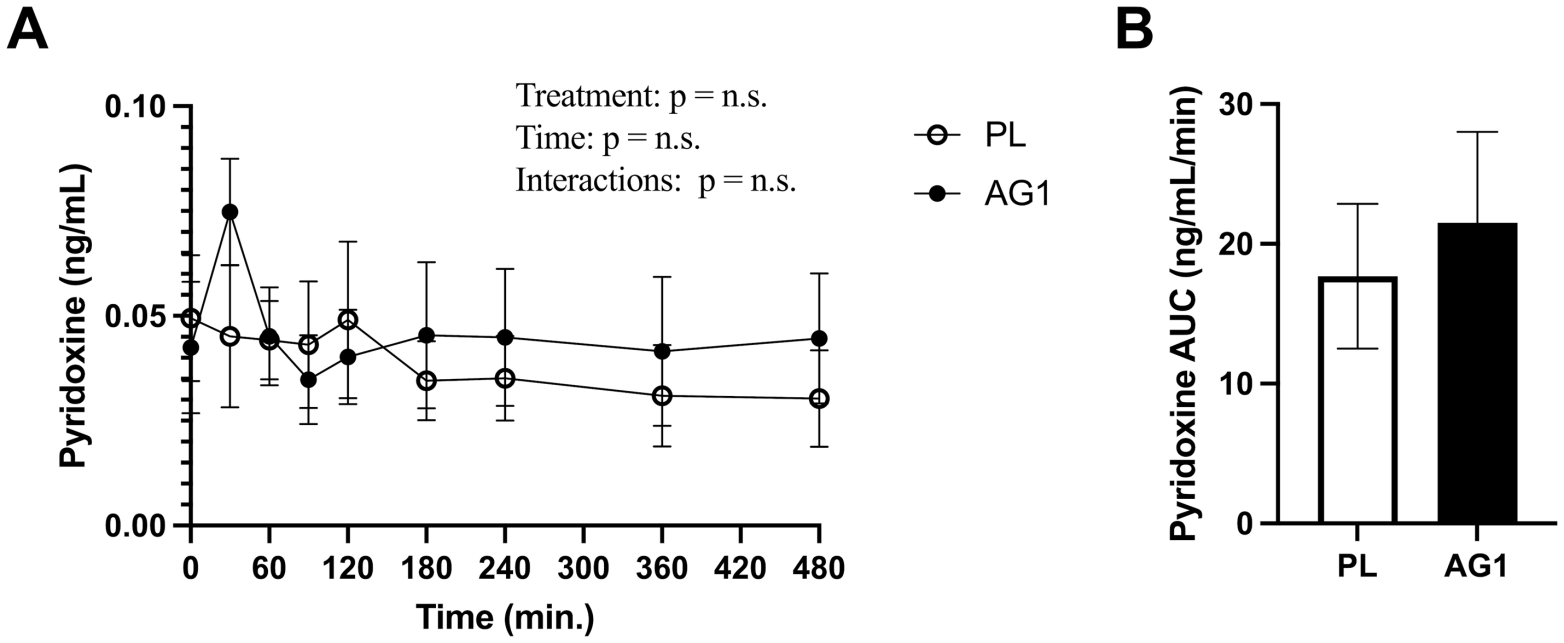
Pyridoxine concentrations and total AUC. Visualization of changes in the pyridoxine curves over the duration of 480 min **(A)** and total AUC **(B)**. ng/mL, nanogram/milliliter; ng/mL/min, nanogram/milliliter/min; and PL, placebo. Data presented as mean ± SEM. Total AUC comparisons were performed using Wilcoxon signed-rank test; *n* = 16.

Pyridoxine (Figures 10A,B and Supplementary Table S5) concentrations were significantly elevated at 30 min post ingestion for AG1 relative to placebo. When AG1 was ingested AUC_0-480 min_ and C_max_ were 21.5 ± 26.0 ng/mL/min and 0.10 ± 0.07 ng/mL, respectively, compared with 17.7 ± 20.7 ng/mL/min and 0.07 ± 0.09 ng/mL following placebo ingestion. The within-subject median difference in AUC_0-480 min_ between AG1 and placebo was 4.0 ng/mL/min (Wilcoxon signed-rank test, *p* = 0.1205) and C_max_ was significantly higher following AG1 ingestion (*p* = 0.0215). T_max_ following AG1 ingestion was 58.1 ± 84.5 min. Sensitivity analyses using iAUC_0-480 min_ yielded similar results (Supplementary Table S5).

#### 3.2.9 Nicotinamide

**Figure 11:**
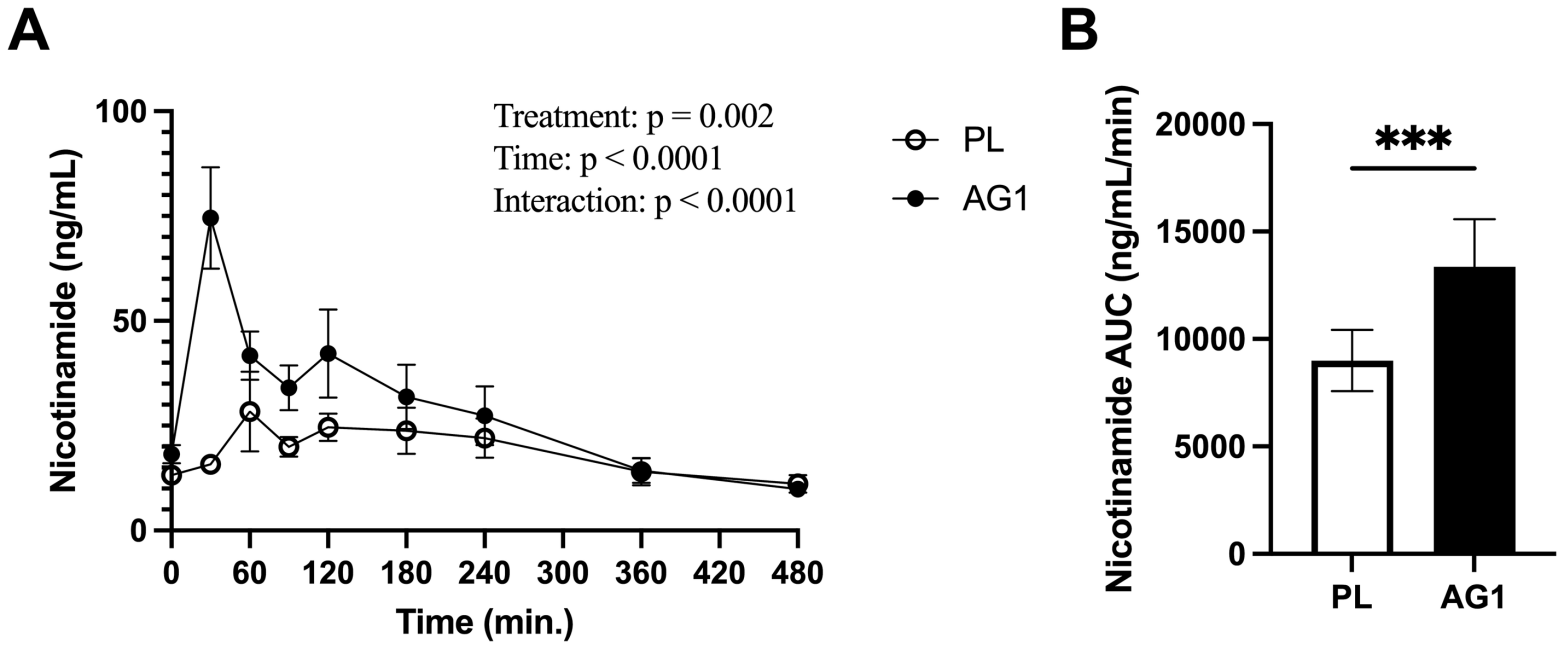
Nicotinamide concentrations and total AUC. Visualization of changes in the nicotinamide curves over the duration of 480 min **(A)** and total AUC **(B)**. ng/mL, nanogram/milliliter; ng/mL/min, nanogram/milliliter/min; and PL, placebo. Data presented as mean ± SEM. ***p < 0.001; *n* = 16.

Nicotinamide (Figures 11A,B and Supplementary Table S5) concentrations were significantly elevated at 30, 60, 90, and 120 min post ingestion for AG1 relative to placebo. When AG1 was ingested AUC_0-480 min_ and C_max_ were 13,358 ± 8,871 ng/mL/min and 81.6 ± 52.5 ng/mL, respectively, compared with 8,994 ± 5,709 ng/mL/min and 38.8 ± 39.8 ng/mL following placebo ingestion. The within-subject mean difference in AUC_0-480 min_ between AG1 and placebo was 4,364 ng/mL/min (95% CI: 2,234, 6,486; *p* = 0.0005) and C_max_ was significantly higher following AG1 ingestion (*p* = 0.0062). T_max_ following AG1 ingestion was 52.5 ± 57.5 min. Sensitivity analyses using iAUC_0-480 min_ yielded differing results with a within-subject mean difference between AG1 and placebo of 1,957 ng/mL/min (95% CI: −716.4, 4,630; *p* = 0.1396) (Supplementary Table S5).

#### 3.2.10 Hesperidin

**Figure 12:**
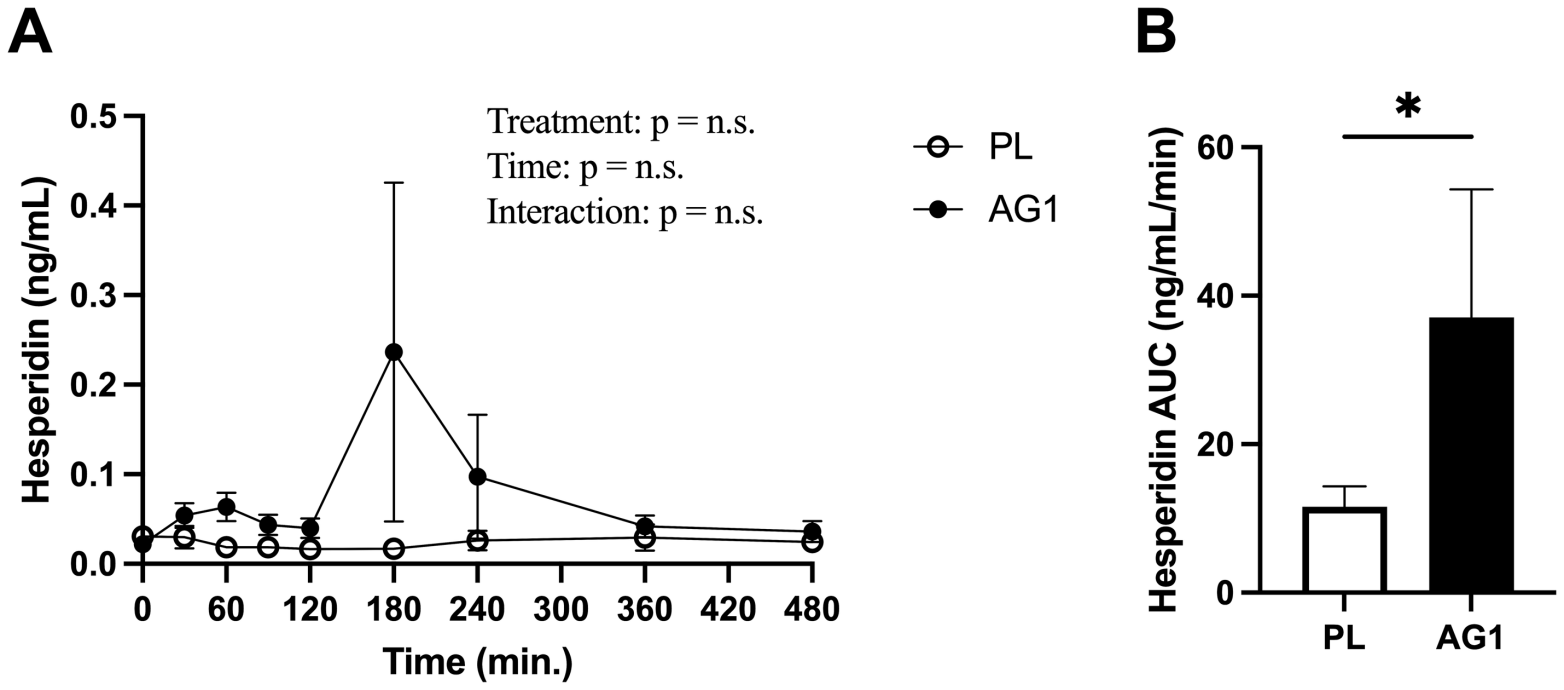
Hesperidin concentrations and total AUC. Visualization of changes in the hesperidin curves over the duration of 480 min **(A)** and total AUC **(B)**. ng/mL, nanogram/milliliter; ng/mL/min, nanogram/milliliter/min; and PL, placebo. Data presented as mean ± SEM. Total AUC comparisons were performed using Wilcoxon signed-rank test. *p < 0.05; *n* = 16.

Hesperidin (Figures 12A,B and Supplementary Table S5) concentration was significantly elevated at 180 min post ingestion for AG1 relative to placebo. When AG1 was ingested AUC_0-480 min_ and C_max_ were 37.1 ± 69.0 ng/mL/min and 0.3 ± 0.8 ng/mL, respectively, compared with 11.6 ± 11.0 ng/mL/min and 0.1 ± 0.7 ng/mL following placebo ingestion. The within-subject median difference in AUC_0-480 min_ between AG1 and placebo was 3.5 ng/mL/min (Wilcoxon signed-rank test, *p* = 0.0323) and C_max_ was not significantly different between conditions (*p* > 0.05). Sensitivity analyses using iAUC_0-480 min_ yielded similar results (Supplementary Table S5).

### 3.3 Tolerability and safety

#### 3.3.1 Tolerability

No significant group x time interactions were observed for any of the VAS items (Table 2). A significant main effect for time and group was observed for hunger ratings (*p* < 0.05). Hunger ratings were higher for the placebo group at 0 and 30 min when compared to AG1 (*p* = 0.005 and *p* = 0.015, respectively).

**Table 2:** Visual analog scale (VAS) ratings following consumption of each intervention.

Variable	Group	0 min	30 min	60 min	90 min	120 min
Bloating (cm)	AG1	1.2 ± 1.2	1.3 ± 1.6	1.4 ± 1.5	1.1 ± 1.2	1.0 ± 1.0
	PL	1.0 ± 1.2	1.5 ± 1.9	1.6 ± 2.0	1.4 ± 1.7	0.9 ± 1.1
Flatulence (cm)	AG1	1.3 ± 1.5	1.3 ± 1.5	1.1 ± 1.2	1.1 ± 1.3	1.1 ± 1.5
	PL	1.3 ± 1.9	1.3 ± 1.8	1.8 ± 2.3	1.5 ± 2.3	1.0 ± 1.6
Hunger (cm)^*^	AG1	3.4 ± 2.2	3.5 ± 2.4	5.0 ± 2.1^$ *β*^	5.4 ± 2.1^$ β^	5.7 ± 1.8^$ β^
	PL	4.4 ± 2.2	4.5 ± 2.0	5.2 ± 2.5	6.0 ± 2.0^$ β^	6.1 ± 2.1^$ β&^
Appetite (cm)	AG1	3.6 ± 2.3	4.1 ± 2.3	5.0 ± 2.2^$ β^	5.5 ± 2.0^$ β^	5.6 ± 1.8^$^
	PL	4.1 ± 2.3	4.7 ± 2.2	5.4 ± 2.5^$^	6.1 ± 2.0^$ β^	6.2 ± 2.0^$ β^
Fullness (cm)	AG1	3.4 ± 2.5	3.5 ± 2.5	3.2 ± 2.2	3.3 ± 2.3	3.2 ± 2.3
	PL	2.8 ± 2.2	2.7 ± 1.8	2.6 ± 1.5	2.5 ± 1.6	2.4 ± 1.8
Prospective food consumption (cm)	AG1	5.0 ± 2.7	5.1 ± 2.3	5.6 ± 1.9	5.9 ± 1.7^$^	6.2 ± 1.8^$ β^
	PL	5.7 ± 2.2	5.8 ± 2.2	5.7 ± 2.6	6.2 ± 2.3	6.8 ± 2.0
Mood (cm)	AG1	6.8 ± 1.5	6.7 ± 1.4	6.7 ± 1.6	6.8 ± 1.5	7.0 ± 1.2
	PL	7.3 ± 1.1	6.9 ± 1.5	6.6 ± 2.2	6.8 ± 2.2	7.0 ± 1.9
Energy (cm)	AG1	5.7 ± 1.5	5.8 ± 1.5	5.9 ± 1.8	6.2 ± 1.8	6.3 ± 1.6
	PL	5.9 ± 1.8	5.5 ± 1.7	5.5 ± 1.7	5.8 ± 1.8	6.0 ± 1.8
Sleepiness (cm)	AG1	4.1 ± 1.8	3.8 ± 1.7	3.4 ± 1.9	3.3 ± 1.7	3.5 ± 2.0
	PL	4.7 ± 2.2	4.4 ± 2.5	4.1 ± 2.0	4.0 ± 1.9	3.0 ± 1.6

Values are presented as mean ± SD for the total sample (*n* = 16). *Indicates a significant difference between groups (*p* < 0.05). ^$^Indicates a significant difference from 0 min (*p* < 0.05). ^β^Indicates a significant difference from 30 min (*p* < 0.05). ^&^Indicates a significant difference from 60 min (*p* < 0.05).

#### 3.3.2 Diet and safety

Assessment of total calories, protein, fat, or carbohydrates revealed no significant differences between groups (*p* > 0.05) and were similar on both visits (Table 3). No differences in body weight or urine specific gravity were observed (Table 3). No significant difference was observed for systolic or diastolic blood pressure (Table 4). The study personnel observed two moderate adverse events from two separate subjects during the placebo condition of the trial (Supplementary Table S6). One subject regurgitated at approximately 90 min following ingestion of the placebo, likely attributed to the investigational product, and one subject reported vasovagal responses at 0, 30, 90, and 240 min during the placebo condition, likely attributed to the blood draws.

**Table 3:** Dietary intake, urine specific gravity, and bodyweight at baseline.

Variable	Group	Mean ± SD
Average calories (kcal)	AG1	1889 ± 715
	PL	1925 ± 645
CHO (g)	AG1	200 ± 91
	PL	197 ± 65
Fat (g)	AG1	83 ± 42
	PL	87 ± 40
Protein (g)	AG1	90 ± 45
	PL	91 ± 43
USG	AG1	1.0177 ± 0.0086
	PL	1.0176 ± 0.0082
Body weight (kg)	AG1	75.4 ± 11.1
	PL	75.5 ± 11.3

Values are presented as mean ± SD for the total sample (*n* = 16). g, grams; kcal, calories; kg, kilograms; PL, placebo; and USG, urine specific gravity.

**Table 4:** Blood pressure and heart rate responses over 8-h post treatment administration.

Variable	Group	0 min	60 min	120 min	180 min	240 min	360 min	480 min
SBP (mmHg)	AG1	117.3 ± 9.4	113.4 ± 10.2	117.1 ± 9.7	115.3 ± 8.9	115.0 ± 10.2	114.2 ± 12.5	113.9 ± 11.2
	PL	116.9 ± 10.0	109.3 ± 10.7	114.9 ± 10.9	115.9 ± 8.4	112.8 ± 8.8	113.5 ± 11.1	114.6 ± 9.5
DBP (mmHg)	AG1	71.8 ± 7.1	72.0 ± 9.4	69.1 ± 9.8	69.3 ± 10.1	70.0 ± 8.9	69.2 ± 11.7	68.8 ± 9.8
	PL	71.8 ± 10.5	68.9 ± 8.8	69.8 ± 9.7	70.3 ± 9.7	66.9 ± 9.5	66.3 ± 9.5	69.2 ± 11.0
HR (bpm)	AG1	63.9 ± 11.9	62.0 ± 10.0	60.3 ± 8.7	58.5 ± 8.8	62.5 ± 9.4	64.3 ± 8.9	65.6 ± 9.2
	PL	63.6 ± 12.4	62.6 ± 11.6	62.4 ± 9.9	61.6 ± 12.5	60.4 ± 9.9	66.2 ± 12.5	63.7 ± 12.5

Values are presented as mean ± SD for the total sample (*n* = 16). BPM, beats per minute; DBP, diastolic blood pressure; min, minutes; mmHg, millimeters of mercury; PL, placebo; and SBP, systolic blood pressure.

## 4 Discussion

This randomized, double-blind, placebo-controlled crossover trial demonstrates that acute ingestion of AG1, which contains vitamins, minerals, and other nutrients, results in measurable systemic absorption of several vitamins, minerals, and phytochemicals. Compared to placebo, AG1 significantly elevated plasma concentrations following consumption and overall exposure (AUC₀-₄₈₀) for multiple nutrients, including folate, vitamin C, calcium, zinc, biotin, nicotinamide, and several B-vitamins, without any observed adverse events or tolerability concerns. These findings confirm that complex, multi-ingredient nutritional formulations can deliver physiologically relevant levels of bioactives to the bloodstream, satisfying the fundamental prerequisite for systemic health.

Absorption and bioavailability of nutrients may be influenced by nutrient-nutrient (18) and nutrient-matrix (e.g., delivery format, texture, etc.) interactions (19). Vitamin C, for instance, is known to enhance non-heme iron absorption by reducing ferric iron to its more soluble ferrous form in the duodenum (30), while minerals like calcium and zinc can compete for absorption via shared transporters at the brush border membrane (31). Important to note, interactions between iron and vitamin C may also be related to gastric vitamin C secretions, which are dependent on the individual’s vitamin C status. These synergistic and antagonistic interactions are well-documented, yet they become increasingly complex as the number of co-ingested nutrients grows. Prebiotics, probiotics, and plant-based fibers are components that may further modulate nutrient bioavailability by altering intestinal pH, enzyme activity, or microbiome composition (32, 33). These matrix effects underscore the importance of empirical data on nutrient pharmacokinetics within multi-ingredient formulations, as theoretical predictions based on single-nutrient data may not reflect real-world bioavailability.

Among the primary nutrients assessed in the present study, folate and vitamin C displayed the most consistent and robust pharmacokinetic profiles. Plasma concentrations rose significantly from baseline and remained elevated for several hours, resulting in substantial increases in both the peak serum concentrations and overall systemic exposure. These results align with findings from prior studies demonstrating efficient absorption and sustained circulation of liposomal vitamin C and non-liposomal formulations (16, 17). Important to note, sequence analyses suggested differences in the treatment effect magnitude between sequences for vitamin C. However, the direction of this difference was not consistent with the pattern expected for a standard carryover effect. This finding likely reflects variability between sequences rather than persistent vitamin C exposure from the prior treatment period. For the additional primary outcomes, calcium and zinc, peak plasma concentrations were observed later (between 120–180 min for calcium, between 60–240 min for zinc), with elevated levels persisting up to 240 min post-ingestion. Notably, levels of these minerals declined to baseline by 360 min contrasting the more sustained profiles seen with folate and vitamin C. Despite these temporal differences, both minerals exhibited significantly higher AUCs and peak concentrations following AG1 consumption compared to placebo. These distinct absorption profiles likely reflect nutrient-specific transport mechanisms such as predominantly active transport for vitamin C, sodium-dependent co-transport for folate, and saturable ion channels for divalent cations like calcium and zinc, as well as potential competition from other nutrients or matrix components. However, in comparison to single ingredient pharmacokinetic data, the present study observed a higher C_max_ and shorter T_max_ than previous work administering the same mineral salts (e.g., calcium citrate, zinc citrate) (34) and various other different mineral salt forms of calcium (e.g., carbonate, pyruvate) and zinc (e.g., acetate, oxide) at doses substantially higher than contained in AG1 (35–38). Although differences in biochemical analysis methods limit direct comparison with previously reported pharmacokinetic data, the more soluble powder format of AG1 likely bypasses the digestive dissociation process, enhancing the bioaccessibility of its constituent nutrients compared to the tablets used in earlier studies (20). Other factors, such as fasted versus fed states, can additionally alter pharmacokinetic parameters (39), underscoring the need for more pharmacokinetic research on nutrients and supplemental products.

Although several statistically significant differences were observed, the clinical relevance of these findings should be interpreted in the context of the nutrient doses in the study product and physiological regulation of the assessed nutrients. With the exception of calcium (9% of the daily value), the nutrients contained in AG1 and assessed in this study are ≥ 100% the daily value, and many of these nutrients are tightly regulated through homeostatic mechanisms that may limit changes in circulating concentrations (40–43). As such, the primary objective of this study was to evaluate absorption from a complex MVM formulation rather than to assess clinical efficacy or long-term nutritional impact.

The absorption kinetics of the exploratory B vitamins varied widely with several vitamins demonstrating significant elevations compared to placebo. Thiamine (B1) showed the broadest temporal profile, with elevated levels from 30 to 360 min. In contrast, pyridoxine (B6) and riboflavin (B2) peaked only at 30 min, with significant increases in AUC_0-480 min_ and C_max_ for riboflavin while pyridoxine demonstrated non-significant increases in AUC_0-480 min_ and significantly elevated C_max_. This discrepancy likely stems from biomarker selection: plasma pyridoxine and riboflavin are less sensitive to acute intake due to rapid conversion to active forms (e.g., pyridoxal 5′-phosphate) or preferential urinary excretion (44, 45). Nevertheless, AG1 produced significant elevations in thiamine, pyridoxine, and riboflavin while administering doses much lower than previously investigated in literature to date (46–50). Future studies may consider urine biomarkers or metabolite profiling to potentially better capture short-term uptake of these vitamins (47). Biotin and nicotinamide demonstrated intermediate absorption kinetics, with significantly elevated plasma levels and AUCs. As biotin is absorbed via both passive diffusion and the sodium-dependent multivitamin transporter (SMVT), potential competition with co-formulated pantothenic acid may influence its bioavailability (51). However, this hypothesis warrants a more targeted subsequent investigation and was outside the scope of this study. Important to note, sensitivity analyses revealed discrepancies in the AUC results for nicotinamide where the AUC_0-480 min_ was significant (*p* = 0.0005) but Δ AUC_0-480 min_ was not significant (*p* = 0.1396). Taken together, this study provides novel, exploratory, evidence that these b-vitamins may have the capacity to significantly elevate blood nutrient levels at supplemental doses lower than previously reported and future work is needed to characterize nutrient pharmacokinetics at doses lower than typically consumed in commercially available single ingredient supplements.

The citrus bioflavonoid hesperidin is included in the AG1 formulation. Given the generally low bioaccessibility and variable absorption of flavonoid glycosides (52), hesperidin was assessed in this study on an exploratory basis. The absorption kinetics demonstrated significantly higher AUC_0-480 min_ compared to placebo and showed a transient but statistically significant elevation at 180 min post-AG1 ingestion, consistent with its presence in the product. Given the recognized challenges of flavonoid bioavailability, particularly for glycoside forms, these preliminary results are encouraging and warrant follow-up studies using additional conjugate-specific assays to better characterize hesperidin-derived bioactivity.

The trial was intentionally conducted in a fasted state, and participants were prompted to repeat the same dietary intake the day before testing in both conditions, to isolate AG1’s absorption profile from dietary confounders. Gastric emptying is typically accelerated under fasting conditions, and this may have contributed to the rapid appearance of certain nutrients in circulation (53). However, the low enzymatic and bile activity during fasting may also impair digestion of fat-soluble or matrix-bound compounds (39, 54). Postprandial hepatic blood flow is also elevated, which may influence nutrient metabolism and first-pass clearance (55, 56). While the fasted state design improves interpretability, it limits generalizability (e.g., supplementation often occurs with meals). Prior studies have shown that fed conditions can either suppress, enhance, or exert a minimal effect on absorption depending on nutrient or pharmaceutical form and food matrix composition (39, 57–60). Thus, future trials should evaluate AG1 in a fed state and compare its performance to single-nutrient reference formulations.

No adverse events were observed or reported for the AG1 treatment group, while two moderate events were reported for the placebo treatment. Additionally, the results of the VAS in this trial did not reveal any significant adverse effects of AG1 on gastrointestinal symptoms (e.g., bloating and flatulence). Taken together, these data provide evidence that AG1, consumed acutely, is likely safe and well tolerated.

Important to note, this trial has several limitations. Traditional pharmacokinetic studies assess absorption, metabolism, distribution, and excretion whereas this study focused on acute absorption. However, this is appropriate for the assessment of MVM supplements given the intricate nuances of factors such as nutrient homeostasis, sex differences, and other biological factors. Due to the modest sample size, sex and age differences were not evaluated but future studies with larger sample sizes should be conducted to rigorously evaluate sex- and age-specific responses. Furthermore, while the present study measured several key vitamins, minerals, and bioactives, full analysis of every nutrient in AG1 was outside the scope of this study. Additionally, while nutrient–nutrient antagonism was not formally evaluated, future studies should include co-analysis of inhibitors such as phytate and oxalate to characterize matrix effects more fully. Baseline vitamin D status was not assessed, which is a determinant of intestinal calcium absorption through its regulation of calcium-binding proteins and active transcellular transport (61). Inter-individual variability in vitamin D status may therefore have contributed to variability in the observed calcium absorption response, and future studies should include baseline vitamin D measurements. Lastly, although the data do not indicate the standardized meal provided at 4 h (included for participant comfort) materially impacted the absorption kinetics of the studied nutrients, it may have influenced later timepoints through its micronutrient content, effects on gastric emptying, or potential interactions affecting nutrient absorption.

## 5 Conclusion

This study provides the first controlled pharmacokinetic evidence that the vitamins, minerals, and bioactives in a complex powdered MVM formulation (AG1) are absorbed into systemic circulation in physiologically meaningful amounts after a single dose for an acute period of time. Key micronutrients such as folate, vitamin C, calcium, and zinc displayed robust plasma appearance and favorable AUC profiles, indicating effective bioavailability. These findings fill a critical gap in the literature, which has historically focused on single-nutrient supplements, and offer a foundation for future research evaluating nutrient delivery, status, and clinical outcomes from complex multi-ingredient formulations.

## Data Availability

The original contributions presented in the study are included in the article/Supplementary material, further inquiries can be directed to the corresponding author.
