# Supplementary material for: Absorption kinetics of vitamins and minerals from a novel nutritional product in physically active adults: a randomized, double-blind, placebo-controlled crossover trial
Source: Front Nutr. 2026 Jul 6;13:1793264. doi: 10.3389/fnut.2026.1793264 (PMC13384010; doi:10.3389/fnut.2026.1793264)
Supplement: Supplementary file 1 [file Supplementary_file_1.DOCX]

**Table S1.** Nutritional information and ingredients for AG1^®^ (Lot #: 02150038). ^††^Measured using viability digital PCR (dPCR), a method that quantifies intact and metabolically active probiotic cells.

|  | **Supplement Facts** | | | |  |
| --- | --- | --- | --- | --- | --- |
|  | **Serving Size: 1 Level AG1^®^ Scoop^ (~ 1 ½ tbsp) (13g)** | |  |  |  |
|  | **Servings per Container: 30** | |  |  |  |
|  |  |  |  |  |  |
|  |  |  | **Amount Per Serving** | **%DV** |  |
|  |  |  |  |  |  |
|  | Calories | | 40 |  |  |
|  | Total Fat | | 1 g | 1%** |  |
|  | Total Carbohydrate | | 6 g | 2%** |  |
|  |  | Dietary Fiber | 2 g | 7%** |  |
|  |  | Total Sugars | <1 g | † |  |
|  | Protein | | 2 g |  |  |
|  | Vitamin A (as beta-carotene) | | 550 mcg RAE | 61% |  |
|  | Vitamin C (as ascorbic acid) | | 500 mg | 556% |  |
|  | Vitamin E (as mixed tocopherols) | | 20 mg | 133% |  |
|  | Thiamin (Vitamin B1) (as thiamine hydrochloride) | | 3 mg | 250% |  |
|  | Riboflavin (Vitamin B2) | | 2 mg | 154% |  |
|  | Niacin (as nicotinic acid, niacinamide) | | 16 mg NE | 100% |  |
|  | Vitamin B6 (as pyridoxal-5-phosphate (P5P)) | | 5 mg | 294% |  |
|  | Folate (as 5-MTHF) | | 680 mcg DFE | 170% |  |
|  | Vitamin B12 (as methylcobalamin) | | 400 mcg | 16667% |  |
|  | Biotin (as D-biotin) | | 330 mcg | 1100% |  |
|  | Pantothenic acid (Vitamin B5) (as calcium pantothenate) | | 4 mg | 80% |  |
|  | Choline (as choline bitartrate) | | 25 mg | 5% |  |
|  | Calcium (as calcium citrate) | | 120 mg | 9% |  |
|  | Iron | | 1 mg | 6% |  |
|  | Phosphorus | | 130 mg | 10% |  |
|  | Magnesium (as dimagnesium malate, magnesium glycinate) | | 30 mg | 7% |  |
|  | Zinc (as zinc citrate) | | 20 mg | 182% |  |
|  | Selenium (as selenized yeast) | | 22 mcg | 40% |  |
|  | Copper (as copper gluconate) | | 0.2 mg | 22% |  |
|  | Manganese (as amino acid chelate) | | 0.4 mg | 17% |  |
|  | Chromium (as chromium picolinate) | | 25 mcg | 71% |  |
|  | Molybdenum (as molybdenum glycinate) | | 45 mcg | 100% |  |
|  | Sodium | | 40 mg | 2% |  |
|  | Potassium (as dipotassium phosphate) | | 250 mg | 5% |  |
|  |  |  |  |  |  |
|  | Vitamin K2 (as menaquinone-7) | | 90 mcg | † |  |
|  | Benfotiamine | | 25 mg | † |  |
|  | Myo-Inositol | | 100 mg | † |  |
|  | Boron (as boron glycinate) | | 500 mcg | † |  |
|  | Coenzyme Q10 (as ubiquinone) | | 60 mg | † |  |
|  | Alpha-lipoic acid (ALA) | | 100 mg | † |  |
|  | Active Superfood and Prebiotic Complex | | 7.5 g | † |  |
|  |  | Organic apple powder, pea protein isolate, organic spirulina, lecithin (>65% phospholipids), inulin (chicory root) powder, citrus bioflavonoids extract, organic chlorella powder, ginger root powder, cocoa bean powder, licorice root powder, bromelain, beta glucan, artichoke aerial parts (15:1) extract, slippery elm bark powder, rhodiola root (15:1) extract, astragalus root (4:1) extract, cocoa seed extract, organic matcha leaf powder, ashwagandha root (5:1) extract, dandelion whole herb (4:1) extract, eleuthero root (10:1) extract |  |  |  |
|  | Daily Phytonutrient Complex | | 1.5 g | **†** |  |
|  |  | Broccoli powder, papaya fruit powder, beet root powder, organic wheat grass leaf powder, organic alfalfa leaf powder, organic barley leaf powder, carrot root powder, acerola fruit (4:1) extract, rosehip fruit (4:1) extract, pineapple fruit powder, shiitake mushroom powder, reishi mushroom powder, rutin (from *Sophora japonica* bud extract), rosemary leaf (4:1) extract, bladderwrack whole plant powder, wolfberry (*Lycium barbarum*) fruit (4:1) extract, burdock root (4:1) extract, hawthorn berry fruit (10:1) extract, bilberry fruit (100:1) extract, milk thistle seed (70:1) extract, grape seed (120:1) extract |  |  |  |
|  | Dairy-Free Probiotic Blend | | 37 mg (10 Billion Viable Cells^††^) | **†** |  |
|  |  | *Lactobacillus rhamnosus* GG*, Lactobacillus acidophilus* NCFM, *Bifidobacterium lactis* HN019, *Lactobacillus casei* LC-11*, Lactobacillus plantarum* LP-115 |  |  |  |
|  |  |  |  |  |  |
|  | **** Percent Daily Values (DV) are based on a 2,000 calorie diet.** | | |  |  |
|  | **† Daily Value (DV) not established.** | | |  |  |

**Table S2**. Intra-day and inter-day accuracy and precision results

| **Analyte** | **QC Level** | **Nominal Conc. (ng/mL)** | **Intra-day** | | **Inter-day** | |
| --- | --- | --- | --- | --- | --- | --- |
|  |  |  | **Accuracy (%)** | **Precision (CV%)** | **Accuracy (%)** | **Precision (CV%)** |
| **Pyridoxal** | LLOQ | 0.978 | 115% | 11.2% | 108% | 9.5% |
|  | LQC | 3.91 | 105% | 9.2% | 108% | 8.9% |
|  | MQC | 31 | 108% | 15.5% | 109% | 9.9% |
|  | HQC | 1000 | 109% | 4.8% | 104% | 5.3% |
| **Riboflavin** | LLOQ | 1.95 | 104% | 12.9% | 99.1% | 20.9% |
|  | LQC | 3.91 | 105% | 12.0% | 103% | 12.2% |
|  | MQC | 15.6 | 109% | 19.4% | 94.0% | 7.1% |
|  | HQC | 250 | 96.1% | 14.4% | 101% | 9.1% |
| **P5P** | LLOQ | 0.488 | 105% | 6.5% | 113% | 3.2% |
|  | LQC | 3.91 | 97.9% | 4.2% | 92.3% | 3.9% |
|  | MQC | 15.6 | 104% | 4.9% | 94.6% | 4.0% |
|  | HQC | 250 | 90.2% | 11.4% | 87.4% | 4.2% |
| **Pyridoxine** | LLOQ | 0.488 | 91.3% | 19.7% | 96.8% | 13.4% |
|  | LQC | 3.91 | 100% | 3.5% | 104% | 6.2% |
|  | MQC | 15.6 | 101% | 13.1% | 102% | 9.5% |
|  | HQC | 250 | 102% | 0.6% | 93.7% | 9.5% |
| **Nicotinamide** | LLOQ | 0.488 | 113% | 12.1% | 115% | 10.7% |
|  | LQC | 3.91 | 97.8% | 10.6% | 100% | 5.4% |
|  | MQC | 15.6 | 104% | 6.7% | 107% | 5.8% |
|  | HQC | 250 | 91.6% | 2.6% | 91.6% | 5.4% |
| **Thiamine** | LLOQ | 0.978 | 106% | 17.9% | 102% | 13.4% |
|  | LQC | 3.91 | 94.7% | 12.7% | 90.1% | 7.1% |
|  | MQC | 15.6 | 94.1% | 1.9% | 92.2% | 3.5% |
|  | HQC | 250 | 89.1% | 6.7% | 86.3% | 7.5% |
| **Biotin** | LLOQ | 0.978 | 106% | 15.5% | 106% | 15.1% |
|  | LQC | 3.91 | 113% | 15.6% | 114% | 15.0% |
|  | MQC | 15.6 | 88.5% | 7.1% | 86.8% | 9.6% |
|  | HQC | 250 | 100% | 8.9% | 102% | 10.7% |

**Table S3**. Optimization details for B-vitamins, Biotin, and Hesperidin

|  | **Ion**  **mode** | **Precursor**  **Ion (m/z)** | **Product Ion (m/z)** | **Dwell time** | **Q1 Pre Bias (V)** | **CE** | **Q3 Pre Bias (V)** |
| --- | --- | --- | --- | --- | --- | --- | --- |
| Thiamine | + | 264.8 | 122.05 | 10 | -30 | -13 | -22 |
| Pyridoxine | + | 170.3 | 152.1 | 20 | -12 | -21 | -24 |
| Pyridoxine-d3 | + | 172.9 | 155.1 | 10 | -21 | -15 | -29 |
| Nicotinamide | + | 123.1 | 80.0 | 10 | -24 | -22 | -15 |
| Nicotinamide-d4 | + | 126.9 | 84.0 | 10 | -24 | -22 | -14 |
| Riboflavin | + | 377.1 | 243.0 | 30 | -30 | -25 | -25 |
| Riboflavin-d7 | + | 384.2 | 250.1 | 10 | -30 | -26 | -27 |
| Biotin | + | 244.9 | 227.1 | 10 | -13 | -14 | -25 |
| Hesperidin | + | 611.0 | 303.1 | 25 | -28 | -25 | -21 |

m/z, mass to charge ratio; V, voltage

**Table S4**. Clinical chemistry and hematological biomarkers at baseline.

| **Variable** | **Mean ± SD** |
| --- | --- |
| WBC (x10E3/uL) | 6.0 ± 2.5 |
| RBC (x10E6/uL) | 4.8 ± 0.5 |
| Hemoglobin (g/dL) | 14.0 ± 1.6 |
| Hematocrit (%) | 41.8 ± 4.0 |
| Glucose (mg/dL) | 90.8 ± 5.0 |
| BUN (mg/dL) | 13.6 ± 4.8 |
| Creatinine (mg/dL) | 1.0 ± 0.2 |
| BUN/Creatinine ratio | 98.5 ± 15.8 |
| eGFR (mL/min/1.73) | 14.7 ± 6.1 |
| Sodium (mmol/L) | 139.3 ± 1.3 |
| Potassium (mmol/L) | 4.4 ± 0.2 |
| Chloride (mmol/L) | 103.3 ± 2.0 |
| CO_2_ (mmol/L) | 23.2 ± 1.8 |
| Calcium (mg/dL) | 9.5 ± 0.4 |
| Total Protein (g/dL) | 7.0 ± 0.4 |
| Albumin (g/dL) | 4.4 ± 0.3 |
| Globulin (g/dL) | 2.5 ± 0.3 |
| A/G ratio (au) | 1.8 ± 0.2 |
| Bilirubin (mg/dL) | 0.5 ± 0.2 |
| Alkaline Phosphatase (IU/L) | 72.8 ± 22.7 |
| AST (IU/L) | 18.3 ± 6.3 |
| ALT (IU/L) | 20.6 ± 9.7 |
| Total Chol (mg/dL) | 173.2 ± 39.8 |
| Triglycerides (mg/dL) | 68.2 ± 25.4 |
| HDL (mg/dL) | 57.8 ± 10.3 |
| VLDL (mg/dL) | 13.3 ± 3.8 |
| LDL (mg/dL) | 102.1 ± 36.6 |
| LDL/HDL | 1.8 ± 0.7 |
| Total/HDL | 3.0 ± 0.7 |

Values are presented as mean ± SD for the total sample (n = 16). A/G ratio, albumin-to-globulin ratio; Albumin, serum albumin; ALT, alanine aminotransferase; Alkaline Phosphatase, alkaline phosphatase; AST, aspartate aminotransferase; BUN, blood urea nitrogen; BUN/Creatinine ratio, blood urea nitrogen to creatinine ratio; eGFR, estimated glomerular filtration rate; g/dL, grams per deciliter; HDL, high-density lipoprotein; IU/L, international units per liter; LDL, low-density lipoprotein; LDL/HDL, low-density lipoprotein to high-density lipoprotein ratio; mg/dL, milligrams per deciliter; mmol/L, millimoles per liter; RBC, red blood cell count; Total Chol, total cholesterol; Total/HDL, total cholesterol to high-density lipoprotein ratio; VLDL, very-low-density lipoprotein; WBC, white blood cell count; x10³/µL, thousands per microliter; x10⁶/µL, millions per microliter.

**Table S5**. Pharmacokinetic results between conditions for all measured nutrients.

| **Variable** | **AG1** | | **PL** | **P-value** | |  |
| --- | --- | --- | --- | --- | --- | --- |
| **Folate** | | | | | |  |
|  |  |  |  |  |  |  |
| AUC_0-480_ (ng/mL/min) | 8,124 ± 1,297 | | 5,489 ± 1,723 | < 0.0001 | |  |
| iAUC_0-480 min_ (ng/mL/min) | 3,297 ± 754.7 | | 131.3 ± 584.8 | <0.0001* | |  |
| C_max_ (ng/mL) | 20.8 ± 0.7 | | 12.9 ± 3.9 | < 0.0001 | |  |
| T_max_ (min) | 37.5 ± 17.3 | | 174.4 ± 111.3 |  | |  |
| **Calcium** | | | | | |  |
|  |  |  |  |  |  |  |
| AUC_0-480_ (mg/dL/min) | 4,448 ± 116.3 | | 4,366 ± 145.6 | 0.0049* | |  |
| iAUC_0-480 min_ (mg/mL/min) | 94.9 ± 92.4 | | 16.0 ± 97.3 | 0.0146 | |  |
| C_max_ (mg/mL) | 9.5 ± 0.3 | | 9.4 ± 0.3 | 0.0468* | |  |
| T_max_ (min) | 129.4 ± 88.8 | | 228.8 ± 167.2 |  | |  |
| **Zinc** | | | | | |  |
|  |  |  |  |  |  |  |
| AUC_0-480_ (μg/dL/min) | 41,140 ± 2,938 | | 35,688 ± 3,353 | < 0.0001 | |  |
| iAUC_0-480 min_ (μg/dL/min) | 4,060 ± 3,423 | | -1,304 ± 50,89 | 0.0011 | |  |
| C_max_ (μg/mL) | 111.6 ± 14.0 | | 87.1 ± 8.8 | < 0.0001 | |  |
| T_max_ (min) | 150.0 ± 68.0 | | 104.0 ± 88.5 |  | |  |
| **Vitamin C** | | | | | |  |
|  |  |  |  |  |  |  |
| AUC_0-480_ (mg/dL/min) | 598.4 ± 276.5 | | 357.7 ± 205.5 | < 0.0001 | |  |
| iAUC_0-480 min_ (mg/mL/min) | 226.4 ± 103.8 | | 0.7 ± 49.6 | <0.0001* | |  |
| C_max_ (mg/dL) | 1.6 ± 0.7 | | 0.9 ± 0.5 | < 0.0001 | |  |
| T_max_ (min) | 146.3 ± 96.0 | | 135.0 ± 144.5 |  | |  |
| **Biotin** | | | | | |  |
|  |  |  |  |  |  |  |
| AUC_0-480_ (ng/mL/min) | 404.4 ± 298.3 | | 154.0 ± 225.1 | < 0.0001 | |  |
| iAUC_0-480 min_ (ng/mL/min) | 263.2 ± 173.1 | | 12.91 ± 135.0 | 0.0009 | |  |
| C_max_ (ng/mL) | 2.3 ± 1.9 | | 0.6 ± 0.7 | 0.0004* | |  |
| T_max_ (min) | 58.1 ± 31.9 | | 138.8 ± 180.6 |  | |  |
| **Nicotinamide** | | | | | |  |
|  |  |  |  |  |  |  |
| AUC_0-480_ (ng/mL/min) | 13,358 ± 8,871 | | 8,994 ± 5,709 | 0.0005 | |  |
| iAUC_0-480 min_ (ng/mL/min) | 4,617 ± 8,164 | | 2,660 ± 6,749 | 0.1396 | |  |
| C_max_ (ng/mL) | 81.6 ± 52.5 | | 38.8 ± 39.8 | 0.0062 | |  |
| T_max_ (min) | 52.5 ± 57.5 | | 116.3 ± 64.7 |  | |  |
| **Pyridoxine** | | | | | |  |
|  |  |  |  |  |  |  |
| AUC_0-480_ (ng/mL/min) | 21.5 ± 26.0 | 17.7 ± 20.7 | | | 0.1205* |  |
| iAUC_0-480 min_ (ng/mL/min) | 1.1 ± 8.8 | -6.1 ± 22.5 | | | 0.0554* |  |
| C_max_ (ng/mL) | 0.1 ± 0.07 | 0.07 ± 0.09 | | | 0.0215* |  |
| T_max_ (min) | 58.1 ± 84.5 | 65.6 ± 69.5 | | |  |  |
| **Riboflavin** | | | | | |  |
|  |  |  |  |  |  |  |
| AUC_0-480_ (ng/mL/min) | 5,054 ± 7,711 | 3,253 ± 5,626 | | | 0.0003* |  |
| iAUC_0-480 min_ (ng/mL/min) | 3,269 ± 6,321 | 1,282 ± 5,110 | | | <0.0001* |  |
| C_max_ (ng/mL) | 19.6 ± 25.7 | 15.3 ± 40.9 | | | 0.0076* |  |
| T_max_ (min) | 78.8 ± 82.6 | 205.6 ± 143.2 | | |  |  |
| **Thiamine** | | | | | |  |
|  |  |  |  |  |  |  |
| AUC_0-480_ (ng/mL/min) | 3,832 ± 3,503 | 730.1 ± 771.9 | | | < 0.0001* |  |
| iAUC_0-480 min_ (ng/mL/min) | 3,046 ± 2866 | -228.0 ± 541.1 | | | <0.0001* |  |
| C_max_ (ng/mL) | 17.4 ± 13.0 | 2.3 ± 2.5 | | | < 0.0001* |  |
| T_max_ (min) | 84.4 ± 49.3 | 174.4 ± 160.0 | | |  |  |
| **Hesperidin** | | | | | |  |
|  |  |  |  |  |  |  |
| AUC_0-480_ (ng/mL/min) | 37.1 ± 69.0 | 11.6 ± 11.0 | | | 0.0323* |  |
| iAUC_0-480 min_ (ng/mL/min) | 26.7 ± 70.7 | -3.2 ± 19.4 | | | 0.0250* |  |
| C_max_ (ng/mL) | 0.3 ± 0.8 | 0.1 ± 0.7 | | | 0.3755* |  |
| T_max_ (min) | 129.4 ± 144.4 | 121.9 ± 150.8 | | |  |  |

Values are presented as mean ± SD for the total sample (n = 16). AUC_0-480_, area under the curve 0-480 minutes; C_max_, maximum concentration; min, minutes; PL, placebo; T_max_, time to maximum concentration; and iAUC_0-480 min_, baseline-adjusted area under the curve 0-480 minutes. * Indicates Wilcoxon signed-rank test due to non-normality of the residual indicated by the Shapiro-Wilk test. T_max_ is reported descriptively; no inferential statistical analysis was performed.

**Table S6**. Adverse events results.

|  | PL  (n=16) | AG1 (n=16) |
| --- | --- | --- |
| **Severity** |  |  |
| Mild | 0 | 0 |
| Moderate | 2 | 0 |
| Severe | 0 | 0 |
| **Relationship to Study Treatment** |  |  |
| Unlikely | 0 | 0 |
| Possible | 0 | 0 |
| Probable | 1 | 0 |
| **Relationship to Testing Procedures** |  |  |
| Unlikely | 0 | 0 |
| Possible | 0 | 0 |
| Probable | 1 | 0 |
| N/A | 0 | 0 |
| **Body System and AEs** |  |  |
| Gastrointestinal | 0 | 0 |
| Regurgitation | 1 | 0 |
| Cardiovascular | 0 | 0 |
| Vasovagal | 1 | 0 |
| Total Number of Adverse Events Experienced During Study | 2 | 0 |
| Total Number of Subjects Experiencing Adverse Events: n (%) | 2/17 (~11.8%) | 0/16 (0%) |

Values are presented as the total number of participants for the total sample (n = 16).
